# Spatial and temporal detection of ‘***Candidatus*** Liberibacter asiaticus’ in ***Diaphorina citri*** through optimized scouting, sampling, DNA isolation, and qPCR amplification in California citrus groves

**DOI:** 10.1371/journal.pone.0323908

**Published:** 2025-05-12

**Authors:** Nathaniel Ponvert, Frank Byrne, Monique J. Rivera, Timo Rohula, Sandra Olkowski, Heshani De Silva Weligodage, Neil McRoberts, Judith K. Brown

**Affiliations:** 1 School of Plant Sciences, The University of Arizona, Tucson, Arizona, United States of America; 2 Department of Entomology, The University of California, Riverside, Riverside, California, United States of America; 3 Department of Entomology, Cornell University, Cornell AgriTech, Geneva, New York, United States of America; 4 Department of Plant Pathology, The University of California, Davis, Davis, California, United States of America; University of Saskatchewan College of Agriculture and Bioresources, CANADA

## Abstract

Huanglongbing (citrus greening disease) is caused by the bacterium ‘*Candidatus* Liberibacter asiaticus’ (*C*Las) (Alphaproteobacteria) and is one of the most destructive bacterial-vector diseases affecting the citrus industry. The bacterium is transmitted by the Asian citrus psyllid (ACP; *Diaphorina citri*). Early detection in citrus trees is challenging due to uneven distribution of *C*Las throughout the tree and a long pre-symptomatic phase of the disease. Due to these limitations, ACP sampling has been suggested as a more reliable early detection strategy. The objective of this study was to develop and optimize approaches for detecting *C*Las in ACP adults and nymphs collected in citrus groves in California using real-time quantitative PCR (qPCR) and droplet digital PCR (ddPCR). The goal was to establish the optimal number of ACP adults and nymphal instar life stages (stages 1–2, 3, or 4–5) that yielded the most reliable detection of *C*Las (Cq values ≤ 38). Results indicated that *C*Las detection correlated with psyllid developmental stage, with the 4th–5th instar nymphs (sample size of five to ten per tube) or adult ACP (sample size of three to ten per tube) providing the most consistent qPCR detection. While *C*Las detection rates increased with adult ACP age, nymphs were preferred for field sampling as adult ACP might have dispersed from non-infected trees, potentially misrepresenting the grove’s *C*Las status. Detection by droplet digital PCR confirmed the presence and genome copies of *C*Las in a subset of ACP across life stages. In field populations, detection rates in nymphs were consistent or stable throughout the year, whereas *C*Las detection in adults exhibited seasonal variation, with *C*Las detection and genome load peaking in January. These targeted ACP sampling strategies and optimized laboratory processing methods will facilitate *C*Las detection in psyllids for streamlining citrus greening disease management.

## Introduction

Citrus greening disease (Huanglongbing) was first reported in Central Asia, India, and South Africa before spreading to citrus-producing regions worldwide [1–3] (reviewed in [4] and [5]). Initially, the disease was believed to result from nutrient deficiency or viral infection. Eventually, the disease was linked to a bacterium ultimately identified as a gram-negative bacterium, “*Candidatus* (*Ca*.) Liberibacter” [6–8]. Three bacterial species have been associated with the disease, “*Ca.* Liberibacter africanus” (*C*Laf), endemic to Africa, “*Ca.* Liberibacter americanus” (*C*Lam) from South America, and ‘*Candidatus* Liberibacter asiaticus’ (*C*Las), native to Asia [9,10]. *C*Laf, The *C*Lam, and *C*Las species are transmitted by the African citrus psyllid *Trioza erytreae* (Del Guercio, 1918) (Hemiptera: Triozidae) and Asian citrus psyllid (ACP) *Diaphorina citri* (Kuwayama, 1907) (Hemiptera: Liviidae). The *C*Lam and *C*Laf species are considered heat-sensitive, and *T. erytreae,* tolerates colder temperatures, by comparison, *C*Las and ACP thrive over the broadest geographical range, and so have caused extensive damage to citrus production [11–13].

Tree infection with *C*Las leads to substantial loss of fruit yield and quality, and eventually leads to tree decline and death [14,15]. ACP acquires *C*Las during feeding as either a nymph or adult, but acquisition as a nymph results in significantly higher pathogen accumulation in the psyllid vector, and a higher risk of future transmission. Therefore, nymphal acquisition is credited with the majority of transmission events [16,17]. Acquisition of *C*Las by ACP occurs from the tree on which they hatch, and nymphs remain on the same tree until they reach maturity [1]. After ingestion, *C*Las multiplies in the midgut cells of ACP and crosses the midgut epithelial barrier to enter the hemolymph and finally access the salivary glands from where the bacterium is most likely transmitted to citrus plants during psyllid feeding in plant phloem [18–21].

To the present date, only one hybrid citrus variety has been identified with natural resistance to citrus greening disease. Most abatement strategies rely on routine chemical pesticide applications to reduce the population load of ACP, and removal of infected trees, both of which are not economically and environmentally sound [15,22,23]. Implementing detection of *C*Las in ACP sentinels to identify potentially infected citrus trees will help mitigate negative economic and environmental effects caused by citrus greening disease outbreaks by providing a tool to detect the presence of *C*Las in groves more rapidly.

Disease spread has been shown to be positively correlated with psyllid vector infestations or foci, which initiate at the edges of citrus groves, particularly on the windward side [5,24–26]. Effective abatement requires standard sampling protocols for accurate surveillance and mapping of the expanding edges of citrus greening disease infection zones.

Understanding the epidemiology of the *C*Las-citrus host pathosystem and early detection of the presence and prevalence of citrus greening disease will rely on the application of economically feasible, meaningful sampling strategies, and sufficient diagnostic power and consistency of sensitive detection. It is proposed here that early-*C*Las detection in sentinel ACP will overcome two main challenges remaining for these goals. First, the long pre-symptomatic period associated with citrus greening complicates symptom-based visual diagnosis of citrus plots, as the population of infected, pre-symptomatic plants persists after the symptomatic plants are identified and removed, leading to ongoing infection from latent *C*Las positive trees [5,27]. Second, the relative load of *C*Las can vary with time of year and between tissue location/type on citrus trees, leading to inconclusive and/or inconsistent molecular detection results for samples collected from citrus leaf tissues [28,29].

The primary objective of this study was to identify optimal sampling strategies for the detection of *C*Las in nymphal and adult stages of ACP. To this end, different stages of ACP that had been colony-reared on *C*Las-positive citrus were collected in different numbers for processing. For each collection scheme of known *C*Las-positive psyllids, the rate of false negatives was calculated and average Cq values for *C*Las-specific and ACP-specific genes were determined. The second objective was to identify spatio-temporal patterns in *C*Las detection using ACP sampling. This was accomplished by analyzing field-collected ACP samples for *C*Las presence and accumulation (Cq and genome copy), to determine if the rates of *C*Las detection and/or *C*Las abundance were correlated with collection scheme, collection location, or collection date. The third objective was to compare the use of two highly sensitive detection methods, real-time quantitative polymerase chain reaction (qPCR)- and droplet digital PCR amplification (ddPCR), for *C*Las detection in the ACP instars. To accomplish this, qPCR and ddPCR analyses were carried out with paired ACP samples, and regression analysis was used to determine the relatedness of the results, and the limits of diagnostic power for the two approaches were compared. Finally, the regression equations fit to the paired data were used to estimate *C*Las genome copy/number of cells detectable in the ACP field samples at different times during the citrus growing season.

## Materials and methods

### Asian citrus psyllid colony establishment and maintenance

The *C*Las-positive and CLas-free ACP colonies were established from adult psyllids collected from HLB-infected citrus trees in an experimental orchard located at the Southwest Florida Research and Education Center (SWFREC), the University of Florida, Immokalee, FL (lat. 26.42⁰ N, long. 81.42⁰ W) in 2006. Psyllids were reared on orange-jasmine plants (*Murraya paniculata* [L.] Jack.) by routine serial transfer (8-10 wks.) maintained in an insect-proof cages (45 × 45 × 50 cm) (BioQuip, Rancho Dominguez, CA) at 25 ± 2°C with a 14:10 h (light: dark) photoperiod and relative humidity (RH) of 60-70%.

Psyllids were collected from colonies using a hand-held aspirator and sorted into groups consisting of nymphal instar stages 1-2, 3, and 4-5, and teneral or mature adults separated by abdomen color. For each group, live cohorts consisting of 1, 3, 5, or 10 individuals, respectively, were placed into a 1.5mL Eppendorf tube containing 95% ethanol.

### *Diaphorina citri* collection from field samples

Populations of *D. citri* were collected from commercial citrus groves in Riverside, Ventura, and San Diego in California. Five citrus groves were sampled in each of the three counties, monthly for 24 months. Sampling was carried out for ten randomly selected perimeter tree blocks at each cardinal direction of the citrus grove. Adult ACP were collected using a gas-powered D-Vac vacuum insect collector (Rincon-Vitova Insectaries, 108 Orchard Dr, Ventura, CA 93001). Each tree was aspirated for 30 seconds as the operator moved around the tree. Nymphs were collected from young flush on the same trees and were transferred into 50mL Falcon tubes containing 95% ethanol. At each site, samples were stored in field coolers with ice packs, prior to transportation to UC Riverside for sorting. No permit was needed to receive dead ACP in alcohol. All ACP samples received for analysis were sorted, and placed live in in tubes containing 95% ethanol.

The adult ACP were sorted based on the color of the abdomen, either blue, gray/white, and orange, and placed into microfuge tubes containing 95% ethanol. The nymphal instars were sorted into groups consisting of 1^st^ and 2^nd^, 3^rd^, and 4^th^ and 5^th^ instars, with each tube containing 5 individuals, and 2–3 tubes collected per sampling site. Samples were shipped by courier to the JK Brown laboratory, The University of Arizona, School of Plant Sciences, bi-weekly or monthly, for *C*Las detection.

### Sample preparation

Between July 2021 and May 2023, 2,120 samples containing nymphs and 12,095 samples containing adults were processed. Due to the high volume of samples, screening was divided into a two-phase protocol. First, each sample was processed individually using a single qPCR run. Samples in cohorts that produced at least one positive *C*Las-specific qPCR result from the initial single-run screening were considered potential “hotspots” and were moved forward into the second phase of screening where each of the cohort members was processed in triplicate.

Stage 1–2 nymph ACP were lysed in a Qiagen TissueLyser II, using a single 3mm metal bead. Stage 3, 4–5, and adult ACP were lysed in a Biospec mini Bead beater using 15–20 2mm zirconium oxide beads. After tissue lysis, nucleic acids were extracted from all samples according to current APHIS standards (APHIS Doc. WI-B-T-1–16 Rev. 7) using the Qiagen DNeasy Blood and Tissue Kit (Cat. No. 69582).

### Realtime quantitative PCR amplification

The *C*Las marker Ribonucleotide Reductase (RNR, JQ866409.1) was used for the detection of *C*Las from sample tissue using the following primers: RNR_F: 5’-CATGCTCCATGAAGCTACCC-3’, RNR_R: 5’-GGAGCATTTAACCCCACGAA-3’, RNR_Probe: 5’-FAM/CCTCGAAATCGCCTATGCAC/3IBkFQ-3’. The *D. citri* gene Wingless (WG, LOC103521460) was used as a positive control and was amplified by real time quantitative PCR from sample tissue using the following primers: WG_F: 5’-GCTCTCAAAGATCGGTTTGACGG-3’ WG_R: 5’-GCTGCCACGAACGTTACCTTC-3’ WG_Probe: 5’-HEX/TTACTGACCATCACTCTGGACGC/3IABkFQ-3’. Both genes were assayed simultaneously from the same sample using primers and probes at 10 µ M concentration each. Samples were processed in triplicate with the following cycling parameters: 50°C 2:00min, 95°C 0:20sec, (40x 95°C 0:15sec, 58°C 1:00min) for 40 cycles. Probe fluorescence was automatically monitored at the completion of each cycle. The qPCR amplification was carried out using a BIORAD C1000 Touch thermocycler equipped with a CFX96 optical reaction module (Product ID# 1845096).

### Determining the most optimal collection scheme for *C*Las-positive and negative instars reared in laboratory colonies

To determine what ACP collection scheme resulted in the most consistent and reliable detection of *C*Las, ACP nymphs and adults sampled from the *C*Las-positive colony were subjected to qPCR-amplification detection. The ACPs were collected into cohorts of 1^st^ and 2^nd^ instars, 3^rd^ instars, 4^th^ and 5^th^ instars, and adults. For each cohort, three replicate samples of 1, 3, 5, and 10 psyllids were collected. Three independent metrics were used to evaluate the consistency of detection between collections. First, the number of false negative results returned from the known *C*Las-positive collections was calculated. Second, the average qPCR Cq values for *C*Las-specific RNR, and ACP-specific WG were compared. Finally, the optimum Cq threshold value that resulted in detection of *C*Las in each sample (groups of 1,3,5, and 10) was determined.

### Droplet digital PCR amplification

*C*Las RNR and ACP WG primers and probes used for ddPCR amplification were the same as those used for qPCR amplification (above). The two genes were quantified simultaneously from the same sample, in duplicate, using primers and probes at 10 µ M concentration each, 2 µ L template volume, and 2x ddPCR supermix (no UTP) (Product ID# 1863024). Droplets were generated using a BIORAD automated droplet generator (Product ID# 1864101). Thermocycling was carried out in a BIORAD C1000 Touch thermocycler (Product ID# 1851196) with a 2°C/second ramp rate, and cycling parameters as follows: 95°C 10:00min, (40x 94°C 0:30sec, 58°C 1:30min) 98°C 10:00min. Droplet quantification was carried out using a BIORAD QX200 Droplet Reader (Product ID# 1864003) and analyzed using the absolute quantification setting in QX Manager Software 2.1.

### Data analysis and visualization

The data from this study were collated into a Microsoft Excel workbook (S1 File). Data analysis, statistics, and figure visualization were performed using MATLABR2023a scripts written by NDP (https://www.mathworks.com/products/matlab.html). For statistical analysis of multiple samples (Figs 2, 3, 8, 10, and S2, S3 and S4 Figs), one-way analysis of variance was implemented, followed by a Bonferroni post-hoc test with a p value of 0.05 for statistical significance. For statistical analysis of two sample types (Fig 9) a two-sided student’s t-test was carried out with a p-value of 0.05 for statistical significance. For determination of the rate of false negative results from the *C*Las positive colony the number of false negative results returned from nine known positive samples was summed and divided by the total number of samples.

**Fig 1:**
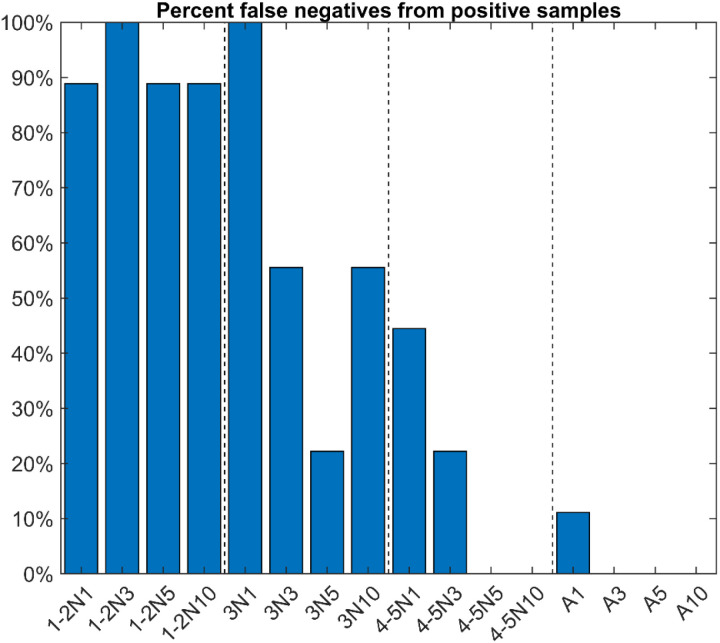
Percent spurious negatives from known ‘*Candidatus* Liberibacter asiaticus’ (*C*Las)-positive samples. For each group, the percentage of spurious negative results returned from nine processed samples was plotted according to the collection group. 1-2N1,3,5,10: Stage 1-2 collected as 1, 3, 5, 10 individuals per tube, respectively. 3N1,3,5,10: Stage 3 collected as 1, 3, 5, 10 individuals per tube, respectively. 4-5N1,3,5,10: Stage 4-5 collected as 1, 3, 5, 10 individuals per tube, respectively. A1,3,5,10: adults collected as 1, 3, 5, 10 individuals per tube, respectively.

**Fig 2:**
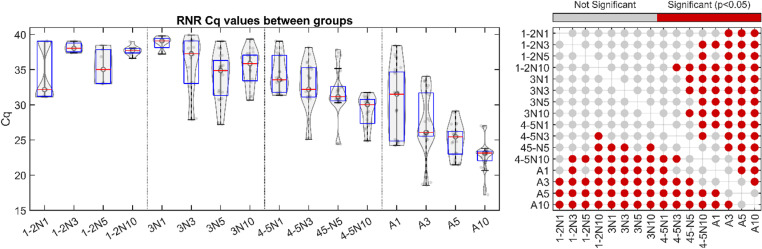
Threshold cycle (Cq) for *C*Las RNR gene detection between groups. For each group, the Cq of the RNR gene is plotted. Grey dots indicate Cq values. Three replicates were processed in triplicate, totaling nine data points. For the box and whiskers, the central mark indicates the median, and the top and bottom edges of the box indicate the 75^th^ and 25^th^ percentile of the data, respectively. Whiskers extend to the most extreme data points not considered outliers. 1-2N1,3,5,10: Stage 1-2 collected as 1, 3, 5, 10 individuals per tube, respectively. 3N1,3,5,10: Stage 3 collected as 1, 3, 5, 10 individuals per tube, respectively. 4-5N1,3,5,10: Stage 4-5 collected as 1, 3, 5, 10 individuals per tube, respectively. A1,3,5,10: adults collected as 1, 3, 5, 10 individuals per tube, respectively. For each grouped collection scheme red dots at right correspond to grouped collection schemes determined to be statistically significantly different using ANOVA with a Bonferroni post-hoc test (p < 0.05).

**Fig 3:**
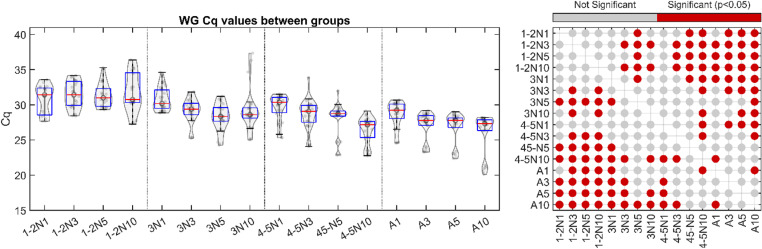
Threshold cycle (Cq) for *D. citri* WG gene target detection between groups. For each group, the Cq of the WG gene is plotted. Grey dots indicate Cq values. Three replicates were processed in triplicate, totaling nine data points. For the box and whiskers, the central mark indicates the median, and the top and bottom edges of the box indicate the 75^th^ and 25^th^ percentile of the data, respectively. Whiskers extend to the most extreme data points not considered outliers. 1-2N1,3,5,10: Stage 1-2 collected as 1, 3, 5, 10 individuals per tube, respectively. 3N1,3,5,10: Stage 3 collected as 1, 3, 5, 10 individuals per tube, respectively. 4-5N1,3,5,10: Stage 4-5 collected as 1, 3, 5, 10 individuals per tube, respectively. A1,3,5,10: adults collected as 1, 3, 5, 10 individuals per tube, respectively. For each grouped collection scheme red dots at right correspond to grouped collection schemes determined to be statistically significantly different using ANOVA with a Bonferroni post-hoc test (p < 0.05).

**Fig 4:**
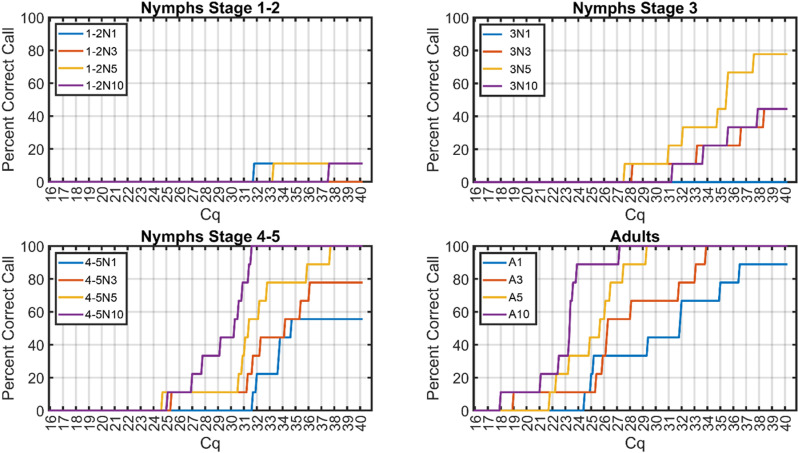
Threshold cutoff values required to correctly call known-positive samples. For each group, the percentage of correctly-called known-“*Ca*. Liberibacter asiaticus” (*C*Las)-positive detection results in samples are plotted against cycle number threshold values between 16 and 40 cycles. As the value increases, the number of known-positive samples that are correctly called as positive, increases. Groups that never reached 100% correctly-called samples are those that produced spurious *C*Las-negative detection results from a known-positive sample.

**Fig 5:**
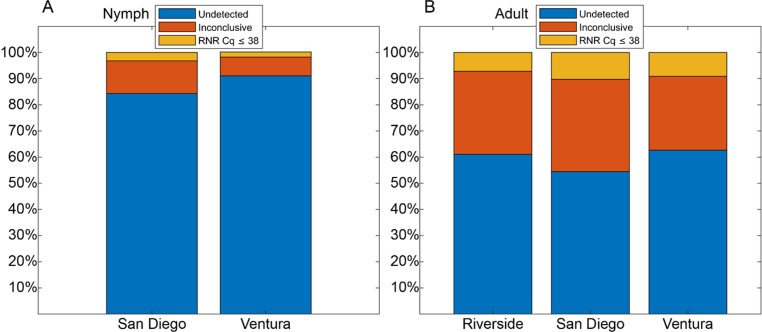
Sample percentage results by collection location. A. Nymphal samples collected in San Diego produced 84.28% negatives (blue), 12.55% samples with RNR Cq between 38 and 40 (orange), and 3.17% “*Ca*. Liberibacter asiaticus” (*C*Las) positive detections (RNR Cq ≤ 38) (yellow). Nymphal samples collected in Ventura produced 91.07% *C*Las-negative detections (blue), 7.15% samples with RNR Cq between 38 and 40 (orange), and 1.98% *C*Las-positive detections (RNR Cq ≤ 38) (yellow). B. Adult samples collected in Riverside produced 61.03% *C*Las-negative detections (blue), 31.8% samples with RNR Cq between 38 and 40 (orange), and 7.18% *C*Las-positive detections (RNR Cq ≤ 38) (yellow). Adult samples collected in San Diego produced 54.44% CLas-negative detections (blue), 35.26% samples with RNR Cq between 38 and 40 (orange), and 10.3% *C*Las-positive detections (RNR Cq ≤ 38) (yellow). Adult samples collected in Ventura produced 62.66% *C*Las-negative detections (blue), 28.27% samples with RNR Cq between 38 and 40 (orange), and 9.07% *C*Las-positive detections (RNR Cq ≤ 38) (yellow).

**Fig 6:**
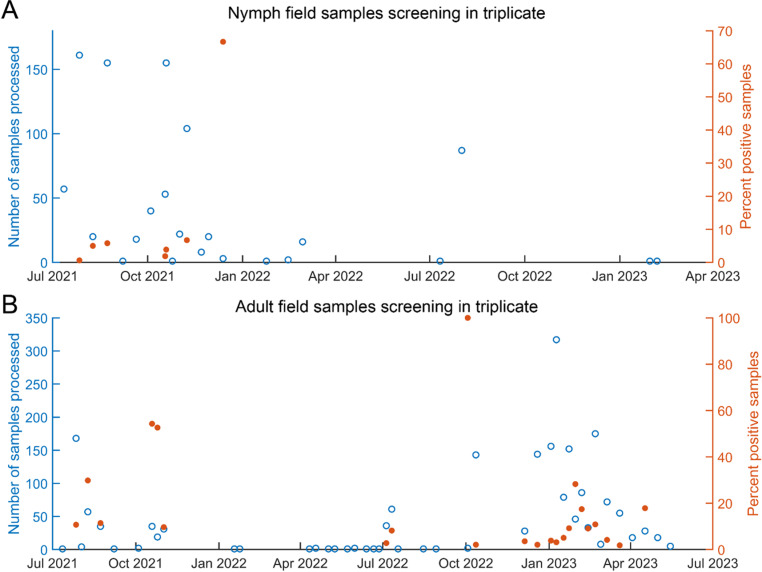
Second phase sample screening as percent positive result by collection date. A. Nymphal samples collected during July 2021 – May 2023 are plotted according to the percent positive rate (orange dots) for a given collection date (total number collected plotted as blue circles). B. Adult samples collected during July 2021 – May 2023 are plotted according to the percent positive rate (orange dots) for a given collection date (total number collected plotted as blue circles).

**Fig 7:**
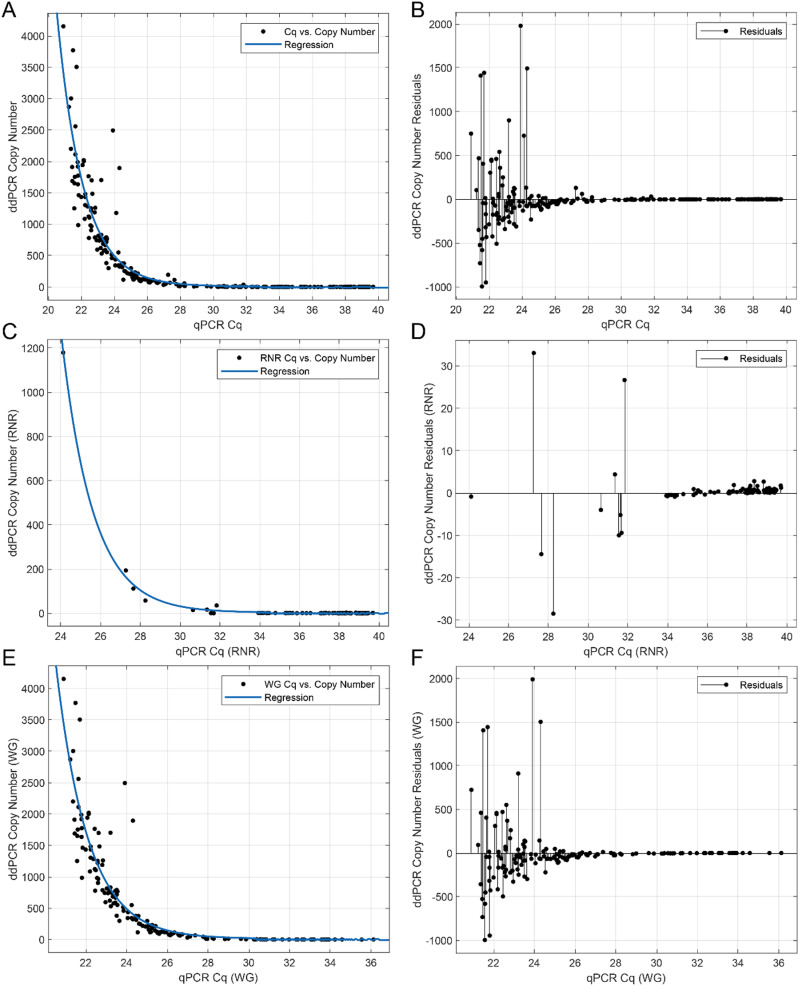
Comparison between Cq and copy number determined using droplet digital PCR amplification. A. The qPCR Cq values and ddPCR copy number collected for all sample types, for RNR and WG target amplification, respectively (black dots, n = 251). Regression curve fit to data, plotted as solid blue line (R^2^ = 0.85748). B. Residuals of regression in panel A with respect to Cq value. C. The qPCR Cq values and ddPCR-determined copy numbers, respectively, for all sample types, for the RNR target amplification (black dots, n = 98). Regression curve fit to data, plotted as solid blue line (R^2^ = 0.99777). D. Residuals of regression in panel C with respect to Cq value. E. The qPCR Cq values and ddPCR copy numbers, respectively, for all sample types, for WG target amplification (black dots, n = 153). Regression curve fit to data, plotted as solid blue line (R^2^ = 0.82792). F. Residuals of regression in panel E with respect to Cq value.

**Fig 8:**
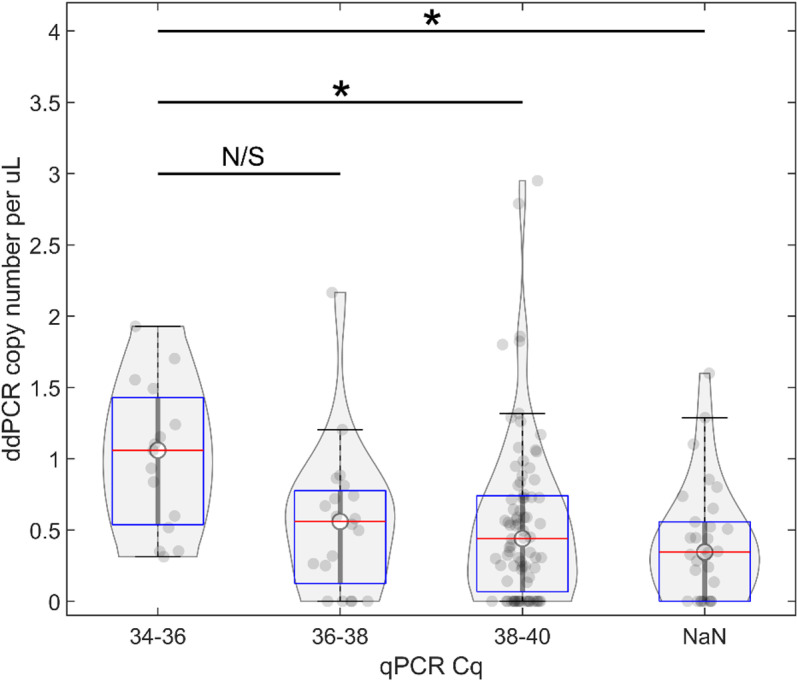
Droplet digital PCR analysis of psyllid samples grouped according to RNR qPCR Cq value ranges. Samples were divided into groups corresponding to their qPCR-derived RNR Cq values. 34-36 (n = 15) and 36-38 (n = 20) represent groups that would be considered *C*Las-positive by qPCR. 38-40 (n = 80) and NaN (n = 30) represent groups that would be considered *C*Las negative by qPCR. ‘*’ = groups with statistically significantly different ddPCR-based template copy number values (ANOVA *F* = 4.17, Bonferroni post-hoc p = 0.0041 and p = 0.0173 for comparisons between group 34-36 and NaN, and group 34-36 and 38-40, respectively).

**Fig 9:**
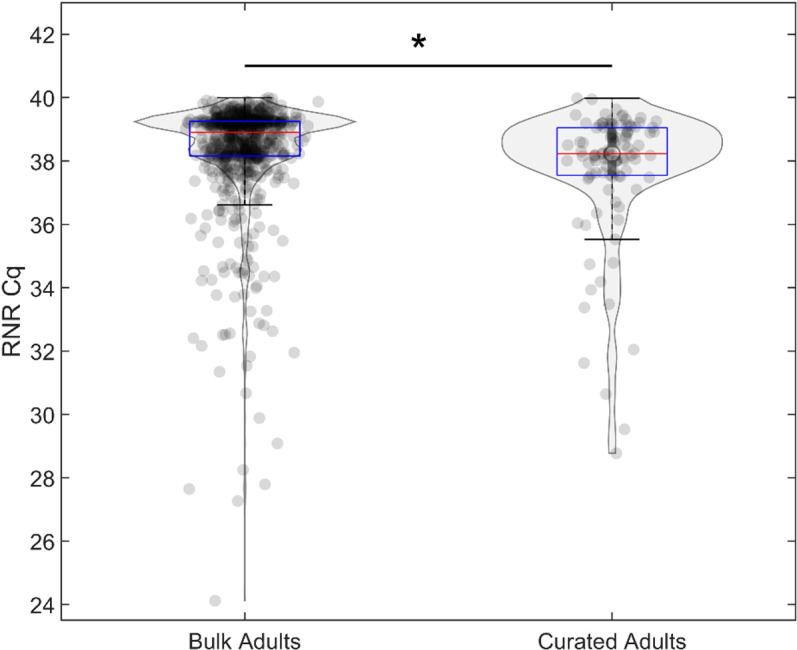
Comparison between ‘*Candidatus* Liberibacter asiaticus’ (*C*Las) RNR Cq values from adults collected on the same date and from the same location as *C*Las-positive nymph samples versus total adult samples. Adult samples with a detectable *C*Las RNR qPCR signal (Cq ≤ 40) were curated for those that had been collected on the same date and from the same location as nymph samples which produced a detectable *C*Las RNR qPCR signal (Cq ≤ 40) (“Curated Adults” n = 105) and compared against the remaining adult samples (“Bulk Adults” n = 1926). The RNR Cq values of adults collected on the same day and from the same location as nymphs are statistically significantly lower than the RNR Cq values of all the adult samples total (mean = 37.7285 and 38.3416, respectively, student’s t-test, p = 0.0013).

**Fig 10:**
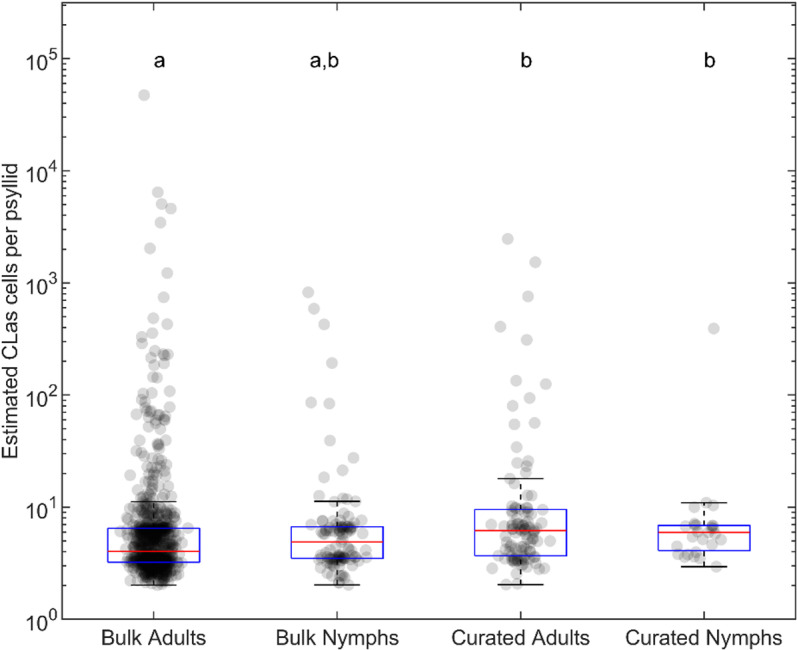
Comparison of ‘*Candidatus* Liberibacter asiaticus’ (*C*Las) cells per psyllid detected in cohorts of adult and nymph samples. Exponential regression as defined in Fig 7C was used to estimate bacterial cell number in bulk adults (n = 1926) bulk nymphs (n = 900), and adults that were collected from the same location on the same date as the *C*Las-positive nymphs (“curated adults”, n = 105, “curated nymphs”, n = 27). “a” and “b” indicate statistically significantly different groups (Kruskal-Wallis ANOVA p = 7.66964e-7).

To calculate the number of *C*Las cells in a representative sample, samples that produced a detectable *C*Las signal by qPCR amplification (RNR Cq < 40) were also analyzed by ddPCR, which assigns a template copy number per reaction value to a given sample [30]. To determine whether paired ddPCR and qPCR results were comparable, the qPCR Cq values and corresponding ddPCR template copy number for tested samples were plotted against one another and a regression line was fitted to the data.

*C*Las cell number was determined from ddPCR template copy number values as follows. The template copy number per µ L was multiplied by 20 (reaction volume in µ L) to calculate the total number of template DNA molecules in the ddPCR reaction. This value was divided by two (number of µ L of template added per reaction) to determine the number of DNA template supplied by each µL of ACP DNA extract added to the ddPCR reaction. This value was multiplied by 100 (total volume of DNA extract from samples in µ L), to determine the total number of templates present in the 100 µ L ACP DNA extract. This number was then divided by 5 to yield the number of templates that were contributed to the eluate by each of the five psyllids from which total DNA was extracted. Finally, this number was divided by five to yield the number of bacterial genomes, based on five copies of RNR known to be present in the *C*Las genome [31].

## Results

### False negative results correlate with sample collection scheme

Stage 1–2 known *C*Las-positive psyllids yielded a high percentage of false negatives regardless of the number collected per tube (Fig 1). The number of false negatives from stage 3 psyllids tended to decrease as more psyllids were collected per tube, but even 10 psyllids per tube collected at stage 3 produced false negative results at low rates. Stage 4–5 psyllids produced no false negatives if at least five nymphs were collected per tube, and adults produced no false negatives if at least three were collected per tube (Fig 1). These results suggest that stage 4–5 psyllids are optimal sentinels if at least five can be collected per tube, or adult samples may be used as sentinels if at least three can be collected per tube. Stage 4–5 nymphs are preferred to adults for use as sentinels, as adult samples may be contaminated with ‘fly-in’ psyllids that do not reflect the *C*Las status of the citrus tree from which they are collected.

### Average *C*Las RNR Cq and ACP WG Cq varies between collection schemes from known *C*Las-positive psyllids

To determine the effect of psyllid stage (instar stage 1–2, stage 3, stage 4–5, or adult) and/or the effect of the number of psyllids collected per sample (1, 3, 5, or 10), the number of qPCR cycles required for each sample to reach fluorescence threshold (Cq) for either the RNR gene or the WG gene was calculated and plotted for each sample type. The Cq for the RNR gene tended to decrease as the age and number of the psyllids collected increased, especially for samples collected from adult psyllids (Fig 2), which may reflect the non-linear increase in numbers of *C*Las cells as the bacterium multiplies throughout the life of the psyllid. The Cq of the WG control decreased consistently with a greater number of psyllids collected per tube, reflective of the stepwise increase in ACP control template concentration in sentinel psyllid samples as a greater number of individuals are available to collect per tube (Fig 3), or possibly to the differences in the efficiency of nucleic acid extraction from ACP adults and/or different ACP nymphal stages (individual or groups). For both genes, statistically significant differences in Cq values between grouped collection schemes are illustrated as a dot plot to the right of the data due to the high number of comparisons between groups.

### Realtime quantitative PCR cycle cutoff threshold value to correctly distinguish known positive samples varies between collection schemes

The USDA-APHIS protocol standardizes the qPCR RNR amplification threshold cycle cutoff value as ≤ 38 to call a sample *C*Las-positive (APHIS Doc. WI-B-T-1–102 Rev. 1). However, the varying average RNR Cq values returned from samples known to be positive (Fig 2) suggests the possibility that the Cq cutoff could be adjusted according to sample type and psyllid collection number to more accurately identify *C*Las positive samples. To determine what RNR threshold cycle number cutoff value is required to correctly call known *C*Las positive samples, the percentage of correctly called known *C*Las-positive samples was plotted against a series of cycle thresholds (16–40 cycles). As the cycle number threshold cutoff increased, the number of correctly called samples for each collection scheme also increased, which is expected, as samples with a Cq less than the threshold are determined to be *C*Las positive. Due to the presence of samples returning false negatives in some of the groups (Fig 1), some sample groups do not return 100% correctly called samples for any cycle threshold cutoff value. For sample types that did not return any false negatives (five stage 4–5 nymphs per tube (4-5N5), ten stage 4–5 nymphs per tube (4-5N10), three adults per tube (A3), five adults per tube (A5), and ten adults per tube (A10)) it was determined that cycle threshold cutoff values of 38, 32, 35, 30, and 28, respectively, were required to correctly call 100% of known-positive samples (Fig 4).

### Analysis of field samples processed between July 2021 and May 2023

For the nymphal samples, 10 out of 2,120 first pass samples produced a positive result, representing cohorts from five collection dates (S1A Fig). The associated samples from the five cohorts were subsequently tested in triplicate, along with several randomly selected samples from outside of the cohorts, totaling 927 samples. For the first phase adult samples, 71 out of 12,095 samples produced a positive result, representing cohorts from seven different collection dates (S1B Fig). The associated samples from the seven cohorts were subsequently tested in triplicate, along with several randomly selected samples from outside of the cohorts, totaling 2,031 samples. The results from these analyses are presented in Fig 6 and S1 Fig.

### Psyllid nymphs collected per tube, and nymph stage compared to sample Cq distribution

Relatively few nymph samples containing fewer than five nymphs per tube were received (96/927 total, Table 1) and to ensure consistency in the analysis of detection rate and comparison between different nymphal instar stages, samples with less than five nymphs per tube were excluded from further analysis, and only samples containing five nymphs per tube were compared between instar stages. The distribution of RNR and WG Cq values for different nymphal instar stages was analyzed and no statistically significant differences between the distribution of RNR Cq values between the different stages was observed (S2 Fig, left, ANOVA *F* = 0.1). In contrast, when the average Cq value of the ACP-specific WG gene was compared between stages, a statistically significant reduction in Cq was observed as the stage of the collected psyllids increased (S2 Fig 2, right, ANOVA *F *= 1745.02), reflecting the increase in the number of ACP cells collected on a per-nymph basis as the stage of the nymph increases. These data reflect that, even as the stage of the ACP nymph increases and the number of ACP nymph cells collected increases, the number of bacterial cells remains relatively constant across nymphal instars.

**Table 1:** Percent positive samples versus the number of nymphs collected per tube.

Nymph Stage	# Nymphs Collected	Total Samples	Positive Samples (RNR Cq ≤ 38)	Percent Positive (%)
Stage 1–2	1	2	0	0
	2	8	0	0
	3	7	1	14.29
	4	7	0	0
	5	345	11	3.19
Stage 3	1	12	0	0
	2	10	1	10
	3	9	0	0
	4	11	1	9.09
	5	247	4	1.61
Stage 4–5	1	13	0	0
	2	8	1	12.5
	3	5	0	0
	4	4	0	0
	5	239	8	3.35

Each collection scheme was divided into total number of samples (column two) and number of positive samples (RNR Cq ≤ 38, column three). The percentage of positive samples for each collection scheme is presented in column four.

### Adult psyllids collected per tube versus percent positive samples and sample Cq distribution

To determine whether there was a correlation between the number of adults collected per tube and the percent of samples that returned a *C*Las positive result, the number of *C*Las positive adult samples was divided by the total number of samples processed for each collection scheme (1, 2, 3, 4, 5, 10 adults per tube). No difference in the percent positive samples per collection scheme was observed (Table 2), except for the samples that contained 10 adults collected per tube. However, only two such samples were collected, making the results Impossible to interpret given the low n value. These data from field collect adult ACP agree with the analysis of known *C*Las positive samples (Figs 1 and 2) where, aside from a single spurious negative from single-adult sample (out of nine total tested), each of the adult collection schemes (1, 3, 5, 10 adults per tube) produced an average Cq that was below the current USDA-APHIS protocol Cq cutoff of 38 cycles. That is, *C*Las-positive adult psyllids produce RNR Cq values consistently below 38 cycles irrespective of the number of adult psyllids collected per tube. Therefore, it is not surprising that the percent positive samples are similar between field samples collected as 1, 2, 3, 4, or 5 psyllids per tube (Table 2). The average RNR and WG Cq values for 1, 2, 3, 4, or 5 adult psyllids per tube collection schemes were also compared. For RNR, there was no statistically significant difference observed between the RNR Cq values between the collection schemes with different numbers of adult psyllids (S3 Fig, left, ANOVA *F* = 0.74), again suggesting that *C*Las positive ACP produce RNR Cq values consistently below 38 cycles regardless of the number collected. For WG, each of the collection schemes was statistically significantly different from each of the others, reflective of the increase in the number of ACP cells collected per sample as the number of psyllids collected increased (S3 Fig, right, ANOVA *F *= 432.48).

**Table 2:** Percent positive samples versus the number of adult psyllids collected per tube.

# Adults Collected	Total Samples	Positive Samples	Percent Positive (%)
1	186	23	12.37
2	147	12	8.16
3	107	12	11.21
4	99	9	9.09
5	1369	121	8.83
10	2	2	100

Each different collection scheme was divided into total number of samples (column two) and the number of positive samples (RNR Cq ≤ 38, column three). The percentage of positive samples for each collection scheme is presented in column four.

### Adult color morph versus rate of *C*Las detection

Average RNR Cq values for adults collected in different numbers per tube were not statistically significantly different (S3 Fig). The relationship between the color morph of field-collected ACP and the rate of *C*Las detection was investigated further to explore whether a particular color morph may be more or less suitable for use as sentinels. When adults were divided into color morphs, no differences were observed between the rate of *C*Las detection between them (Table 3). These data suggest that no particular adult color morph is more suitable as a *C*Las detection sentinel, and supports the rationale for the selection of stage 4–5 psyllids as optimal for *C*Las detection.

**Table 3:** Percent positive samples versus color morph.

Color morph	Total Samples	Positive Samples	Percent Positive (%)
Blue	1226	108	8.81
Orange	264	25	9.47
White	541	46	8.50

Each different color morph was divided into total number of samples (column two) and the number of positive samples (RNR Cq < 38, column three). The percentage of positive samples for each collection scheme is presented in column four.

### Sample collection location compared to percentage of positives

Of the nymphal stage samples tested in triplicate, only two were collected in Riverside County. Both samples yielded RNR Cq values that were detectable but greater than 38, indicating that they were negative for *C*Las. Of the remaining samples, 758 were from citrus groves in San Diego with 119 producing detectable signal for the RNR qPCR reaction, 24 of which reach fluorescence threshold before cycle 38, representing 15.72% and 3.17%, respectively. The remaining 168 samples were received from Ventura County. Among them, 15 produced detectable RNR signal, of which only 3 reached the fluorescence threshold before cycle 38, representing 8.93% and 1.78%, respectively. Therefore, for nymphal samples processed between July 2021 and May 2023, those received from San Diego County were, on average, approximately twice as likely to be positive for *C*Las than those received from Ventura County (Fig 5A).

Of the 2,031 samples containing adult psyllids, 780 were collected in Riverside County, 777 were collected in San Diego County, and 474 were collected in Ventura County. Of the samples from Riverside County that produced detectable signal from the RNR qPCR reaction (248 samples), 56 reached fluorescence threshold intensity in ≤ 38 cycles, representing 31.8% and 7.18%, respectively. Of the samples from San Diego County that produced detectable signal from the RNR qPCR reaction (274 samples), 80 reached fluorescence intensity in ≤ 38 cycles, representing 35.26% and 10.3%, respectively. Of the samples from Ventura County that produced detectable signal from the RNR qPCR reaction (143 samples), 43 reached fluorescence threshold intensity in ≤ 38 cycles, representing 28.27% and 9.07% respectively. Like the data collected/results from samples containing nymphs, adult samples with the highest percent positive rate were collected from San Diego County (Fig 5B), although the difference was less striking in samples containing adults than it was in samples containing nymphs. To assess whether the distribution of Cq values for differing numbers of adults per tube varied by the collection location, the Cq data were split according to their collection location and plotted according to the number of psyllids collected per tube (S4 Fig). There was no difference observed between the distribution of Cq values for any collection scheme between any of the three locations (S4 Fig) (ANOVA *F = *0.35, 0.04, 0.48, 0.91, 0.16 for 1, 2, 3, 4, and 5 psyllids per tube, respectively). While the difference in positive samples observed between collection locations was slight, it is interesting to consider that temperatures in San Diego county remain in the optimal range for ACP throughout the majority of the year, while other sites exhibit greater degrees of temperature fluctuations into suboptimal ranges. These weather patterns may promote the growth and development of ACP beyond the range that is facilitated in other locations (‘N. McRoberts’, unpublished data).

### Sample collection date and the percentage of ‘Candidatus Liberibacter asiaticus’-positive samples

To determine if there was a correlation between sample collection date and the percentage of samples reporting *C*Las positive status, the number of positive samples for a given collection date from the second round of screening were plotted as a percentage of the total number of samples collected on that day. Of the 927 nymph samples analyzed, 137 produced a detectable signal for the RNR qPCR reaction, with 27 producing a signal that reached fluorescence threshold before cycle 38, representing 14.78% and 2.91%, respectively. Of the 927 samples moved into phase two of testing, 819 were collected in 2021, 107 were collected in 2022 and 2 were collected in 2023. All the nymph samples producing a positive *C*Las signal (qPCR RNR Cq ≤ 38) were collected during 2021, probably reflective of the unequal distribution of nymph collections per year. The percentage of samples reporting *C*Las positive status remained relatively steady throughout 2021 (hovering ~5% median rate of positive detections) (Fig 6A). One notable deviation from this trend occurred for samples collected in December of 2021; however, only 3 samples (n = 3) were collected during this period, making these results uninterpretable.

Of the 2,031 samples containing adult psyllids that were moved forward into phase two screening, 835 produced a signal for the RNR qPCR reaction, with 179 producing a signal that reached fluorescence threshold before cycle 38, representing 41.11% and 8.81%, respectively. To assess whether there was a correlation between adult sample collection date and the percentage of samples reporting *C*Las positive, the number of positive samples for a given collection date were plotted as a percentage of the total number of samples collected on that day. Notably, spikes in the rate of percent *C*Las positive samples were observed in the summer of 2021 and winter of 2023 (Fig 6B), and if this trend is found to be consistent year to year, sampling ACP sentinels in these seasons could serve as ‘economical diagnostic indicators’, in lieu of a year-round, random sampling routine which is expensive and time-consuming.

### Comparison between qPCR and ddPCR amplification *C*Las detection assays

To determine whether droplet digital PCR (ddPCR) was comparable to results of qPCR detection and therefore could be used for reliable detection of *C*Las in ACP, field collected samples that were determined to be *C*Las positive by qPCR were also analyzed using ddPCR and the paired results for each sample were compared. Both *C*Las-specific RNR and ACP-specific WG were assessed using ddPCR. The relationship between WG and RNR reaction results using qPCR and ddPCR were assessed in bulk for all sample types (Fig 7A, B), and separately for all sample types (Fig 7C-F). When different psyllid stages were also analyzed individually (S5-S8 Figs) the qPCR-based Cq and ddPCR- copy number values generally agreed with one another, where for both RNR and WG reactions for all sample types together the R^2^ value of the regression line fit to the data was 0.85805 (Fig 7A,B). When the WG and RNR reactions were plotted separately, RNR produced a substantially higher R^2^ value than WG: R^2^ = 0.99779 and R^2^ = 0.82792, respectively (Fig 7C-F). Importantly, most of the variation between qPCR Cq values and ddPCR copy number values for the WG target reaction was observed for samples with relatively low Cq values (<~25), suggesting that an overabundance of DNA template may differentially skew qPCR and ddPCR results. This was consistent with analogous observations for the WG target when psyllid stages were analyzed individually. For stages 1 and 2, stage 3, and stages 4 and 5, and adult ACP cohorts, the R^2^ values for regression lines fit to WG data tended to decrease as the age of the psyllids increased, correlating with on average lower Cq: R^2^ = 0.92907, 0.92635, 0.84816, 0.46302, respectively. (S5C,D, S6C,D, S7C,D, S8C,D Figs). For RNR, the reverse trend was observed, with the R^2^ values of regression lines fit onto the data increasing sharply as the age of the psyllid cohort increased - R^2^ = 0.0071231, 0.012791, 0.024877, 0.99789 for stages 1 and 2, stage 3, and stages 4 and 5, and adult ACPs, respectively. The opposite trend observed between the two examples could reflect a ‘sweet spot’ of DNA concentration for which both qPCR and ddPCR amplification results agree. In cases with an overabundance of DNA template (e.g., WG reactions for stage 4–5 and adult psyllids) the high template concentration could skew the approaches differently, possibly due to the handling of multiple templates per droplet in the ddPCR Poisson distribution calculation of abundance and/or template saturation during qPCR cycling. Conversely, in cases with extremely low DNA template (e.g., RNR reactions for stage 1–2, stage 3, and stage 4–5 psyllids) the low template concentration may skew either type of approach due to fractional recovery during reactions.

### Comparison between real-time quantitative PCR and droplet digital PCR amplification limit-of-detection

As the ddPCR detection assay was explored for the purpose of being used as a diagnostic tool for samples with the potential for having extremely low template concentration (i.e., only a few bacterial cells per psyllid), the variation between the qPCR and ddPCR results in reactions with low template concentration were explored further. To accomplish this, samples were divided according to their qPCR Cqs into groups comprising Cqs between 34–36, 36–38, 38–40, and NaN (40+). After samples were divided into groups, their corresponding ddPCR copy number determinations were plotted. An analysis of variance (ANOVA) was used to determine the Cq at which ddPCR is capable of differentiating samples from those with either NaN (40+) or 38–40 (*C*Las negative by qPCR) Cq values. Samples with a qPCR Cq range of between 34–36 produced ddPCR-based copy number values that were statistically significantly higher than ddPCR-based copy number values returned from NaN-Cq or Cq between 38–40 (*C*Las negative) samples (Fig 8, ANOVA *F* = 4.17, Bonferroni post-hoc p = 0.0041, p = 0.0173, respectively). The median copy number per µ L for samples with Cqs between 34–36 was 1.059, whereas for samples with Cqs between 38–40 and NaN (40+) the median copy numbers per µ L were 0.43945 and 0.34522, respectively. These results suggest that the diagnostic power of ddPCR may be slightly lower than that of qPCR, in that ddPCR is not able to statistically differentiate samples that would be considered negative by qPCR amplification (Cq > 38 or NaN) from samples that would be considered positive by qPCR amplification (Cq between 36–38) (Fig 8). However, a comparison between the qPCR and ddPCR results based on the overall number of samples considered positive (for qPCR meaning a Cq ≤ 38, for ddPCR a copy number per uL > 0) suggests the opposite. From the data shown in Fig 8, 110 of 130 samples showed Cq values of > 38 and would therefore be considered negative by current qPCR validation standards. By comparison, only 33 samples returned a ddPCR copy number values of 0, which could be interpreted to indicate that the ddPCR detection assay was more sensitive than the qPCR assay. Together, these results suggest congruent but not entirely overlapping results between the two methodologies and underscore the need for a standardized metric for what is considered positive and negative by ddPCR, similar to the published qPCR detection threshold guideline that has established the cutoff at 38 cycles.

### Estimation of ‘*Candidatus* Liberibacter asiaticus’ cell load among ACP samples most likely to have inoculated citrus trees that became diseased

While some variation was observed when comparing *C*Las detection by qPCR compared to ddPCR amplification detection, the high R^2^ value returned by the regression line fit to the RNR Cq values (Fig 7C, R^2^ = 0.99779) allowed for the estimation of bacterial cell number detected in cohorts of collected ACP. To investigate the *C*Las cell load associated with cohorts of ACP that are most likely to be actively transmitting citrus greening disease, field samples were curated for collection locations and times from which adult ACP with detectable RNR qPCR signal (qPCR Cq ≤ 40) were collected alongside nymph samples that produced a detectable RNR qPCR signal (qPCR Cq ≤ 40). Among the ACP life stages analyzed in this study, these adult samples most likely to be representative of psyllids that could feasibly have transmitted *C*Las to citrus, given that the ACP nymphs collected from the same trees must have ingested and then acquired *C*Las from feeding on the leaves inoculated with the bacterium by bacteriliferous adults. First, the qPCR Cq values of the adult samples from the curated dataset, e.g., samples collected from the same location at the same time as the *C*Las-positive nymph samples), were compared to the Cq values of the adult samples, minus the curated adults). The results indicated that the RNR Cq values were statistically significantly lower (student’s t-test, p < 0.05) among the adults collected at the same time and from the same location as the *C*Las-positive nymphs, than those of the total adult samples, in that the curated adult RNR Cq and total adult RNR Cq means were 37.7285 and 38.2645, respectively (Fig 9). Notably, the adults from the curated dataset RNR Cq’s values were nearly always lower than 38 while the total adult sample RNR Cqs were most often greater than 38, results that are consistent with the thresholds established in the USDA APHIS protocol or CLas detection in ACP sentinels.

The regression line fit onto the paired RNR qPCR and ddPCR data (Fig 7C) was used to derive the number of bacterial cells detected per sampled adult and nymph psyllids that were collected on the same day from the same location. Using this approach, it was found that adults collected at the same time and location as positive nymphs had a statistically significantly higher estimated number of bacterial cells, as compared with other adults - estimated median *C*Las cell load in bulk samples ~4 *C*Las cells per sample, estimated median cell load in curated samples ~6 *C*Las cells per sample (Fig 10). The relative increase in detected bacterial cell abundance in psyllids that are likely to have transmitted *C*Las agrees with the corresponding decrease in Cq values (Fig 9) and could reflect increased rates of transmission observed in adult psyllids that acquired *C*Las sufficiently early as nymphs to allow the bacterium to multiply and accumulate to high levels.

## Discussion

This study establishes a foundation for the rapid and efficient detection of ‘*Candidatus* Liberibacter asiaticus’ (*C*Las) in citrus groves using *Diaphorina citri* as sentinels or indicators of *C*Las infection in citrus trees. The work outlined here overcomes two main obstacles for accurate and early detection of citrus greening disease in citrus groves: lingering latent infection from pre-symptomatic trees which hinders visual diagnosis, and unequal distribution of *C*Las cells in citrus tissue which hinders molecular diagnosis. First, the results of this study identified the rate of false negatives from known *C*Las-positive samples, which is critical for using ACP as sentinels as psyllids collected at suboptimal life stages may yield inconsistent or unreliable results making them unsuitable for this purpose. Initial analysis of known *C*Las-positive colonies highlighted differences in detection reliability. Results showed that consistent *C*Las detection could be achieved with samples containing either five stage 4–5 nymphs or three to five adult psyllids (Fig 1). Triplicate sample analyses further characterized the distribution of *C*Las RNR and ACP WG qPCR Cq values in known *C*Las-positive samples (Figs 2 and 3). These findings indicated that *C*Las levels disproportionately increase in adult psyllid samples, likely due to *C*Las multiplication during the psyllid’s lifespan. Using the RNR qPCR Cq values established for each sample group, the study determined an optimal threshold value for reliably identifying the presence or absence of *C*Las in different sample types (Fig 4). It was determined that Cq thresholds of 38, 32, 35, 30, and 28 were sufficient to accurately detect RNR in known *C*Las-positive samples comprised of five stage 4–5 nymphs, ten stage 4–5 nymphs, three adults, five adults, and ten adults respectively. These results suggest that while a qPCR cycle threshold of 38 is sufficient, it may not be required for samples of all types. Although it is possible that the bacterial load in processed psyllid samples could be affected by pooling multiple psyllids, the samples were collected and processed in this way to reflect the average bacterial load that could be expected for a given psyllid stage and/or collection location.

Field samples collected from July 2021 to May 2023 were categorized into nymph and adult cohorts. No significant differences in *C*Las-positive detection rates were observed based on collection location (Fig 5). These results may reflect the widespread distribution of *C*Las in all three counties sampled in California. Nymph samples consistently maintained a *C*Las-positive detections rate of approximately 5% (Fig 6A, S1 Fig). No correlation was observed between nymph stage and either *C*Las-positive detections rates or RNR Cq distribution (S2 Fig, Table 1). Taken together, these results suggest a wide distribution of *C*Las, which remains at relatively stable titer across the ACP nymphal stages. Therefore, if nymphs are used as sentinels for CLas detection, the 4^th^ and 5^th^ nymphal stages, which have harbor more CLas. This is likely due to the incremental increase of *C*Las accumulation due to ongoing multiplication in the immature psyllids as they molt into subsequently older nymphal stages.

The adult psyllid samples were associated with a spike in rate of positive *C*Las detections during and shortly after the month of January (Fig 6B), which is consistent with previously published reports of seasonal spikes in *C*Las detection. There was no association between the number of adult psyllids collected per tube and the rate of *C*Las-positive detections (S3 Fig, Table 2). This result was consistent with the observation that known *C*Las-positive adult psyllids yielded robust detection results regardless of the number collected/analyzed per tube, aside from samples consisting of a single psyllid per tube, which were considered to be unreliable because of the small sample size (Fig 1, Fig 4). Further, there was no association between the rate of CLas-positive detections and the number of adult psyllids collected per tube when the samples were divided by collection location (S4 Fig), corroborating the observation from S3 Fig. Taken together these results suggest that if adults are to be collected for detecting *C*Las, samples comprised of at least three adults per tube are preferred, and could be collected in or around January for optimal economic efficiency.

A subset of the field samples were analyzed for the presence of *C*Las using both qPCR and ddPCR amplification assays; to determine the relationship between qPCR Cq and template copy number between reactions (Fig 7, S5-S8 Figs). The ddPCR-based copy number estimation largely agreed with qPCR-based Cq determination (Fig 7), although when high levels of DNA template were present variability among the values was observed (Fig 7, S5-S8 Figs). A comparison of the limits of detection for PCR and ddPCR amplification approaches indicated good overlap in the diagnostic conclusions. However, evidence of some variability between the results produced by two methods highlights the importance of developing standard operating procedures for ddPCR *C*Las detection.

Regression data were used in combination with field data that were curated for *C*Las-positive adult psyllid samples collected from the same location on the same date as nymph samples determined as *C*Las-positive (Figs 9 and 10). These adult samples are the most likely to represent those that had inoculated citrus trees with *C*Las, because the paired nymph samples would have ingested and acquired *C*Las from the leaves on which they hatched, into which *C*Las was inoculated by the adults. The *C*Las RNR Cq values of the curated adult samples were significantly lower than those for the total adult samples, reflective of the high level of bacterial load in potential *C*Las-transmitting adult psyllids (Fig 9).

The regression equation shown in Fig 7C was also used to calculate the number of bacterial cells detected in the paired samples. It was observed that the level of bacterial load was higher in adults that were collected at the same time and from the same location as the positive nymphs compared to the general adult cohort of samples, which was possibly related to a generally higher accumulation of *C*Las in psyllids that are actively transmitting the pathogen (Fig 10). As shown in Fig 8, the diagnostic power of ddPCR could be considered higher than that of qPCR - 110 samples had qPCR-determined RNR Cqs > 38 out of 130 total samples and would therefore be considered negative by current qPCR standards. In comparison, only 33 samples returned ddPCR RNR copy number values of 0. These results suggest that a standardized metric for what is considered positive and negative via ddPCR will need to be developed, similarly to the current guidelines for qPCR setting the cutoff at 38 cycles. The qPCR 38 cycle threshold is congruent with the observation presented in Fig 9, psyllids most likely to be actively transmitting HLB have RNR Cqs < 38, while the bulk collected psyllids have RNR Cqs > 38. Therefore, it is proposed that the ddPCR threshold could be based on this metric, and set at a positive call being determined at ≥ 5 *C*Las cells detected per sample.

In summary, collection of at least five 4^th^ and 5^th^ instar nymphs per tube is preferred for use as *C*Las sentinels because they more reliably reflect the presence or absence of *C*Las in the tree from which they are collected and do not have the potential to be contaminated with ‘fly in’ psyllids from surrounding trees. However, adult psyllids were observed to carry a generally higher load of *C*Las cells and may provide an opportunity for an economic seasonal collection/detection scheme. If adults are to be collected, at least three should be collected per tube to avoid false negatives. The results of this study suggests that psyllids provide a robust, consistent platform for the detection of *C*Las in citrus groves, and that a ‘psyllid-sentinel’ detection approach is amenable to current qPCR detection approaches and next-generation ddPCR-based approaches. This study also suggests that a seasonal detection scheme could be developed to decrease the economic impact of detecting citrus greening disease.

## Supporting information

S1 Fig Initial phase psyllid sample screening as percent “*Candidatus* Liberibacter asiaticus” (*C*Las)-positive detection, by collection date. **A.** Nymphal instars collected during July 2021 – May 2023 are plotted according to the *C*Las-percent positive rate (orange dots) for a given collection date (total number collected, plotted as blue circles). **B.** Adult psyllid samples collected during July 2021 – May 2023 are plotted according to the percent *C*Las-positive rate (orange dots) for a given collection date (total number collected, plotted as blue circles).
(TIF)

S2 Fig RNR and WG Cq distribution for psyllid field samples, separated by nymphal stage. Enough nymph samples containing five nymphs per tube were collected to allow comparison between the distribution of RNR and WG Cq values versus nymph stage at collection. RNR: stages 1 and 2 n = 59, stage 3 n = 28, and stages 4 and 5 n = 32. WG: stages 1 and 2 n = 345, stage 3 n = 247, and stages 4–5 n = 239. Samples with Cq below 38 cycles (stages 1 and 2 n = 11, stage 3, n = 4, and stages 4 and 5, n = 8) were positive for “*Ca*. Liberibacter asiaticus” detection. For the RNR Cq values, no statistically significant difference was observed between the nymph stages (ANOVA *F = *0.1). For the WG Cq values, each stage was statistically significantly different from all of the others (ANOVA *F* = 1745.2, Bonferroni post hoc p < 0.05), denoted by asterisks over line. For RNR/WG ratio values, each stage was statistically significantly different from all of the others (ANOVA *F* = 264.51, Bonferroni post hoc p < 0.05), denoted by asterisks over line.
(TIF)

S3 Fig Adult psyllid field sample Cq distribution compared to the number of psyllids per tube. The distribution of RNR and WG Cq values for field samples of adult psyllids was grouped according to the number of psyllids collected per tube and plotted. 1 psyllid/tube n = 186, 2 psyllids/tube n = 147, 3 psyllids per tube n = 107, 4 psyllids per tube n = 99, 5 psyllids per tube n = 1369. For RNR, no statistically significant difference was observed between any of the Cq values when compared between the number of psyllids collected per tube (ANOVA *F = *0.74). No statistically significant difference was observed between the distribution of the RNR Cq values between the number of psyllids collected per tube (ANOVA *F = *0.74). For the WG Cq values, each of the groups was statistically significantly different from all of the others (ANOVA *F* = 342.48, Bonferroni post hoc p < 0.05), denoted by asterisks over line.
(TIF)

S4 Fig Distribution of RNR Cq values for each collection scheme, separated by collection location. The distribution of Cq values for field samples of adult psyllids that returned *C*Las positive results (RNR qPCR Cq ≤ 38) was divided into the number of psyllids collected per tube and plotted according to the sample collection location. For Riverside County samples: 1 psyllid/tube n = 22, 2 psyllids/tube n = 19, 3 psyllids per tube n = 11, 4 psyllids per tube n = 10, 5 psyllids per tube n = 242. For San Diego County samples: 1 psyllid/tube n = 30, 2 psyllids/tube n = 28, 3 psyllids per tube n = 19, 4 psyllids per tube n = 18, 5 psyllids per tube n = 257. For Ventura County samples: 1 psyllid/tube n = 29, 2 psyllids/tube n = 21, 3 psyllids per tube n = 16, 4 psyllids per tube n = 14, 5 psyllids per tube n = 97. Black line indicates cycle threshold of 38 cycles. Samples with Cq below black line (Riverside County samples: 1 psyllid/tube n = 6, 2 psyllids/tube n = 3, 3 psyllids per tube n = 4, 4 psyllids per tube n = 3, 5 psyllids per tube n = 40. San Diego County samples: 1 psyllid/tube n = 10, 2 psyllids/tube n = 4, 3 psyllids per tube n = 4, 4 psyllids per tube n = 3, 5 psyllids per tube n = 57. Ventura County samples: 1 psyllid/tube n = 7, 2 psyllids/tube n = 5, 3 psyllids per tube n = 4, 4 psyllids per tube n = 3, 5 psyllids per tube n = 24) were determined to be positive. No statistically significant difference was observed between the distribution of Cq values between the number of psyllids collected per tube divided between sample types (ANOVA *F = *1.66, 1.12, 0.24 for Riverside, San Diego, and Ventura Counties, respectively).
(TIF)

S5 Fig Comparison between Cq and copy number determined by digital droplet PCR amplification (ddPCR) for 1st and 2^nd^ nymphal stages. **A**. The Cq values and ddPCR copy number for 1^st^ and 2^nd^ nymphal stages based on RNR target amplification (black dots, n = 12). Regression curve fit to data, plotted as a solid blue line (R^2^ = 0.0071231). **B**. Residuals of regression in panel A with respect to Cq value. **C.** The qPCR Cq values and ddPCR copy numbers, respectively, for 1^st^ and 2^nd^ nymphal stages, based on WG target amplification (black dots, n = 33). The regression curve fit to data, plotted as a solid blue line (R^2^ = 0.92907). **D**. Residuals of regression in panel C with respect to Cq value.
(TIF)

S6 Fig Comparison between Cq and copy number determined by droplet digital PCR amplification (ddPCR) for the 3rd stage psyllid nymphs. **A**. The qPCR Cq values and ddPCR copy numbers, respectively, for the 3^rd^ nymphal stage based on RNR target amplification (black dots, n = 26). Regression curve fit to data, plotted as a solid blue line (R^2^ = 0.12791). **B**. Residuals of regression in panel A with respect to Cq value. **C.** The qPCR Cq values and ddPCR-copy numbers, respectively, for the 3^rd^ nymphal stage, based on WG target amplification (black dots, n = 36). The regression curve fit to data, plotted as a solid blue line (R^2^ = 0.92635). **D**. Residuals of regression in panel C with respect to the Cq values.
(TIF)

S7 Fig Comparison between Cq and copy number determined by droplet digital PCR amplification (ddPCR) for 4^th^ and 5^th^ nymphal stages. **A**. The Cq values and ddPCR copy numbers, respectively, for 4^th^ and 5th nymphal stages based on RNR target amplification (black dots, n = 24). Regression curve fit to data is plotted as a solid blue line (R^2^ = 0.024877). **B**. Residuals of regression in panel A with respect to the Cq values. **C.** The qPCR Cq values and ddPCR-determined copy numbers, respectively, for the psyllid 4^th^ and 5th nymphal stages, based on WG target amplification (black dots, n = 39). The regression curve fit to data is plotted as a solid blue line (R^2^ = 0.84816). **D**. Residuals of regression in panel C with respect to the Cq values.
(TIF)

S8 Fig Comparison between Cq and copy number determined by droplet digital PCR amplification (ddPCR) for psyllid adults. **A**. The Cq values and ddPCR copy numbers, respectively, for adult psyllids based on RNR target amplification (black dots, n = 36). Regression curve fit to data, plotted as a solid blue line (R^2^ = 0.99789). **B**. Residuals of regression in panel A with respect to the Cq values. **C.** The Cq values and ddPCR copy numbers, respectively, for adult psyllids based on WG target amplification (black dots, n = 45). Regression curve fit to data, plotted as a solid blue line (R^2^ = 0.46302). **D**. Residuals of regression in panel C with respect to the Cq values.
(TIF)

S1 File Raw data collated into Microsoft Excel file format, used, analyzed, and presented in this study. (XLSX)
