# Supplementary figures and images for: Spatial and temporal detection of ‘Candidatus Liberibacter asiaticus’ in Diaphorina citri through optimized scouting, sampling, DNA isolation, and qPCR amplification in California citrus groves
Source: PLoS One. 2025 May 12;20(5):e0323908. doi: 10.1371/journal.pone.0323908 (PMC12068614; doi:10.1371/journal.pone.0323908)

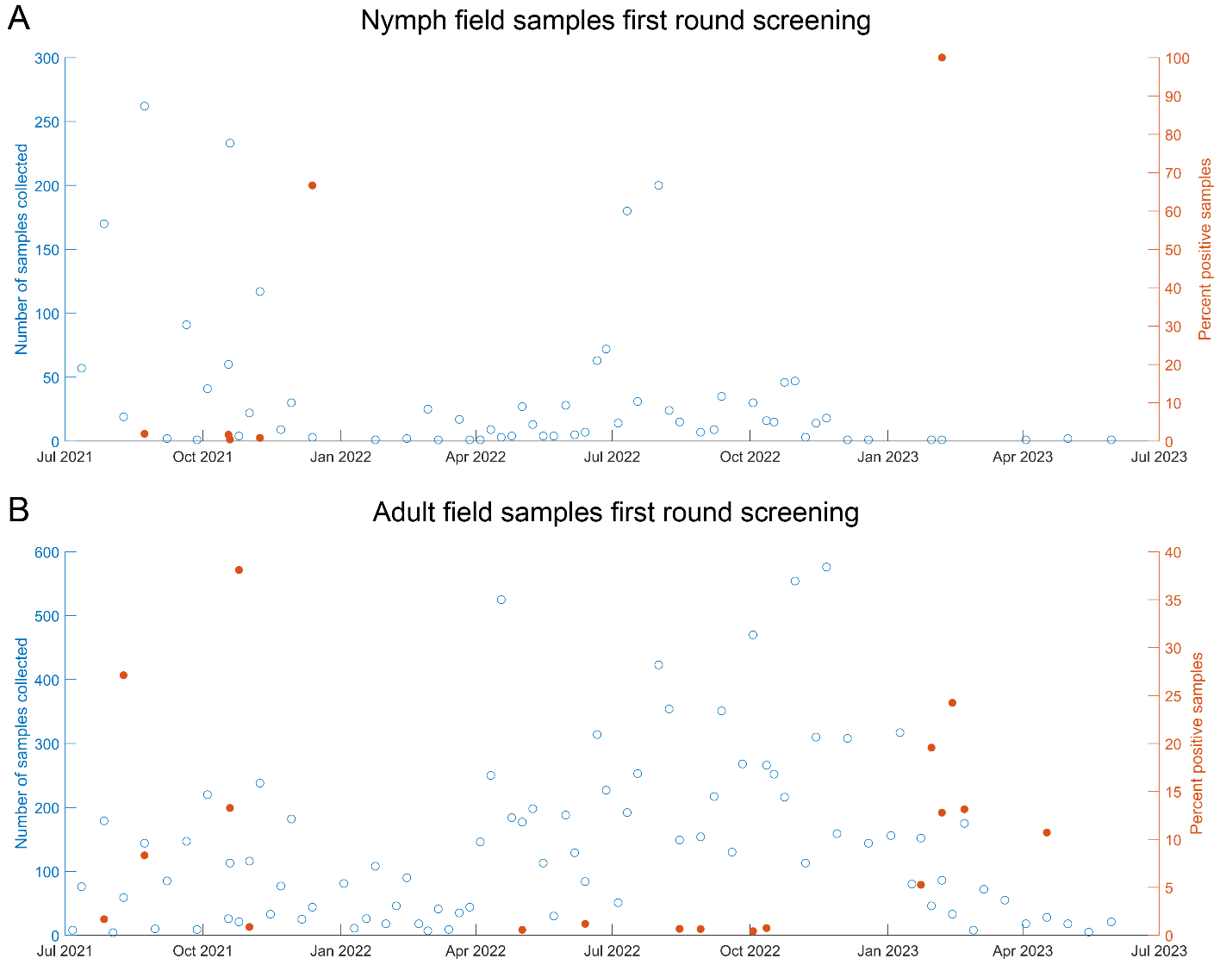

Supplement: S1 Fig — A. Nymphal instars collected during July 2021 – May 2023 are plotted according to the CLas-percent positive rate (orange dots) for a given collection date (total number collected, plotted as blue circles). B. Adult psyllid samples collected during July 2021 – May 2023 are plotted according to the percent CLas-positive rate (orange dots) for a given collection date (total number collected, plotted as blue circles). (TIF) [file pone.0323908.s001.tif]

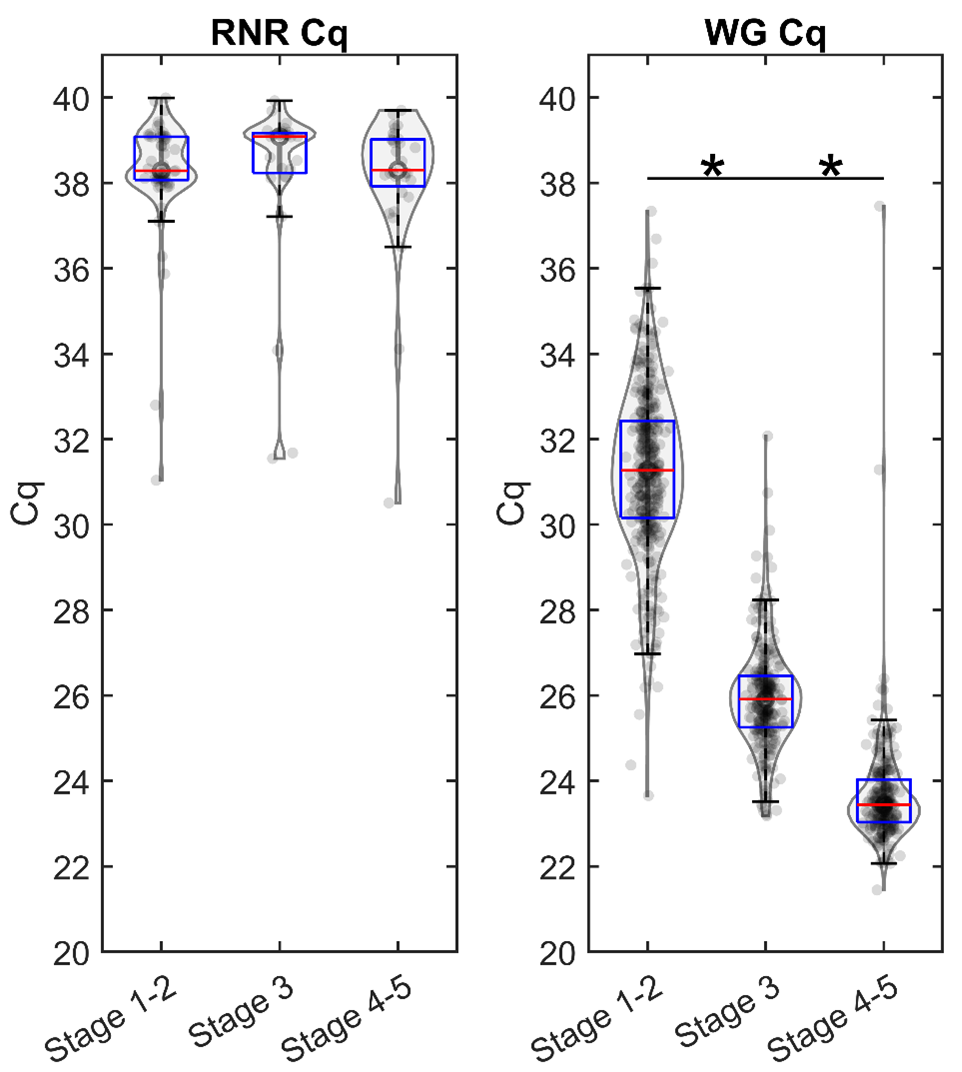

Supplement: S2 Fig — Enough nymph samples containing five nymphs per tube were collected to allow comparison between the distribution of RNR and WG Cq values versus nymph stage at collection. RNR: stages 1 and 2 n = 59, stage 3 n = 28, and stages 4 and 5 n = 32. WG: stages 1 and 2 n = 345, stage 3 n = 247, and stages 4–5 n = 239. Samples with Cq below 38 cycles (stages 1 and 2 n = 11, stage 3, n = 4, and stages 4 and 5, n = 8) were positive for “Ca. Liberibacter asiaticus” detection. For the RNR Cq values, no statistically significant difference was observed between the nymph stages (ANOVA F = 0.1). For the WG Cq values, each stage was statistically significantly different from all of the others (ANOVA F = 1745.2, Bonferroni post hoc p < 0.05), denoted by asterisks over line. For RNR/WG ratio values, each stage was statistically significantly different from all of the others (ANOVA F = 264.51, Bonferroni post hoc p < 0.05), denoted by asterisks over line. (TIF) [file pone.0323908.s002.tif]

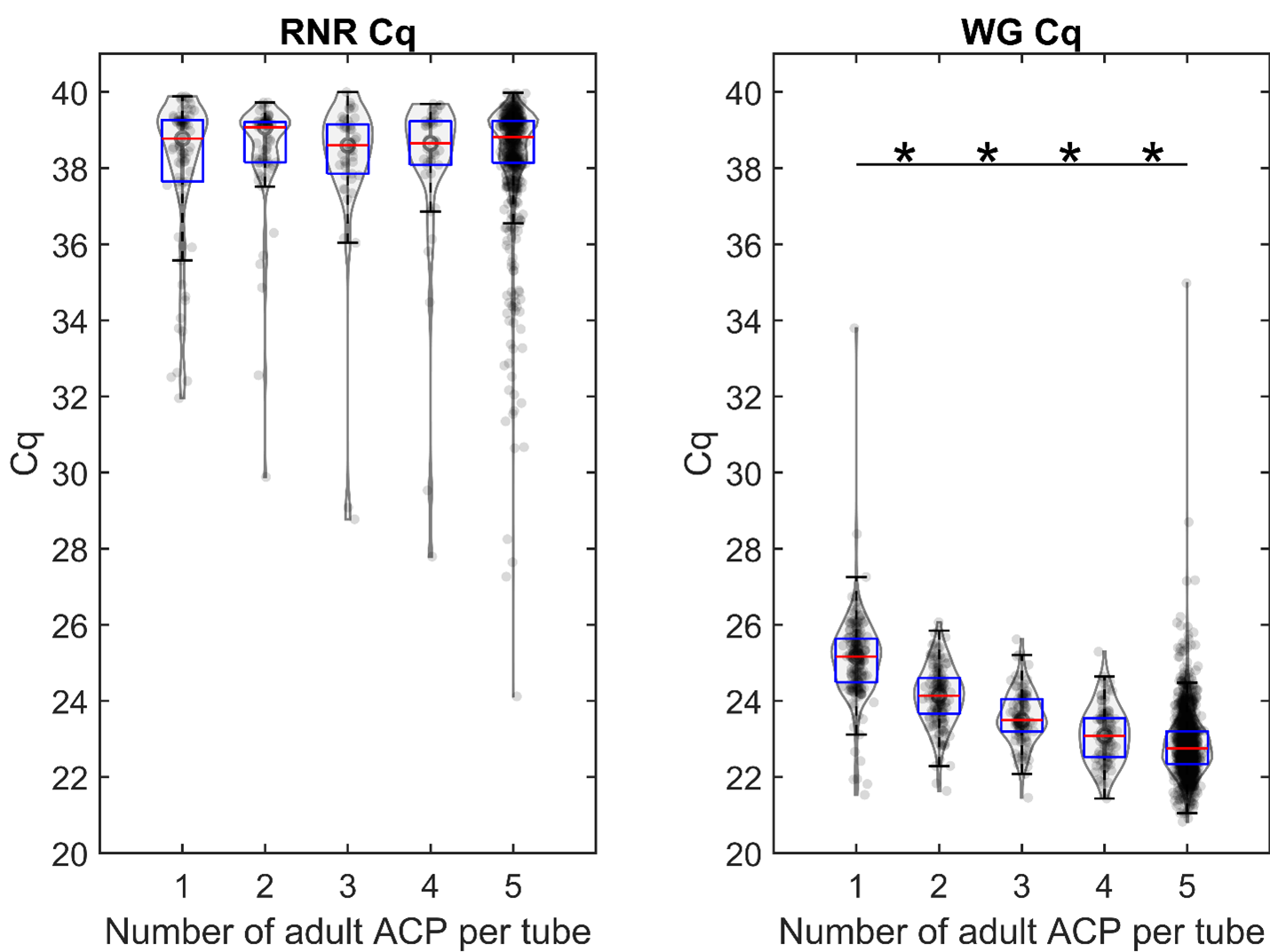

Supplement: S3 Fig — The distribution of RNR and WG Cq values for field samples of adult psyllids was grouped according to the number of psyllids collected per tube and plotted. 1 psyllid/tube n = 186, 2 psyllids/tube n = 147, 3 psyllids per tube n = 107, 4 psyllids per tube n = 99, 5 psyllids per tube n = 1369. For RNR, no statistically significant difference was observed between any of the Cq values when compared between the number of psyllids collected per tube (ANOVA F = 0.74). No statistically significant difference was observed between the distribution of the RNR Cq values between the number of psyllids collected per tube (ANOVA F = 0.74). For the WG Cq values, each of the groups was statistically significantly different from all of the others (ANOVA F = 342.48, Bonferroni post hoc p < 0.05), denoted by asterisks over line. (TIF) [file pone.0323908.s003.tif]

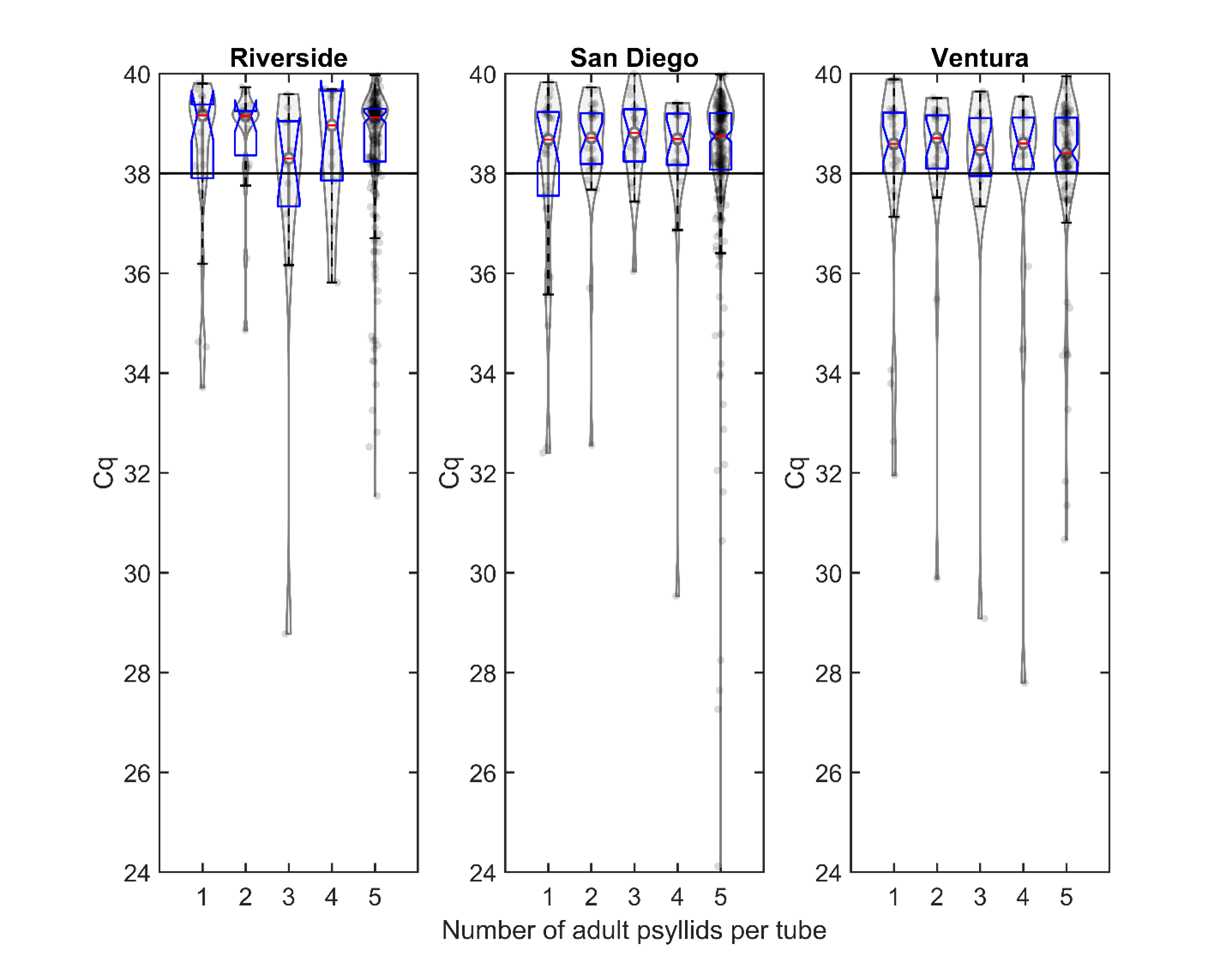

Supplement: S4 Fig — The distribution of Cq values for field samples of adult psyllids that returned CLas positive results (RNR qPCR Cq ≤ 38) was divided into the number of psyllids collected per tube and plotted according to the sample collection location. For Riverside County samples: 1 psyllid/tube n = 22, 2 psyllids/tube n = 19, 3 psyllids per tube n = 11, 4 psyllids per tube n = 10, 5 psyllids per tube n = 242. For San Diego County samples: 1 psyllid/tube n = 30, 2 psyllids/tube n = 28, 3 psyllids per tube n = 19, 4 psyllids per tube n = 18, 5 psyllids per tube n = 257. For Ventura County samples: 1 psyllid/tube n = 29, 2 psyllids/tube n = 21, 3 psyllids per tube n = 16, 4 psyllids per tube n = 14, 5 psyllids per tube n = 97. Black line indicates cycle threshold of 38 cycles. Samples with Cq below black line (Riverside County samples: 1 psyllid/tube n = 6, 2 psyllids/tube n = 3, 3 psyllids per tube n = 4, 4 psyllids per tube n = 3, 5 psyllids per tube n = 40. San Diego County samples: 1 psyllid/tube n = 10, 2 psyllids/tube n = 4, 3 psyllids per tube n = 4, 4 psyllids per tube n = 3, 5 psyllids per tube n = 57. Ventura County samples: 1 psyllid/tube n = 7, 2 psyllids/tube n = 5, 3 psyllids per tube n = 4, 4 psyllids per tube n = 3, 5 psyllids per tube n = 24) were determined to be positive. No statistically significant difference was observed between the distribution of Cq values between the number of psyllids collected per tube divided between sample types (ANOVA F = 1.66, 1.12, 0.24 for Riverside, San Diego, and Ventura Counties, respectively). (TIF) [file pone.0323908.s004.tif]

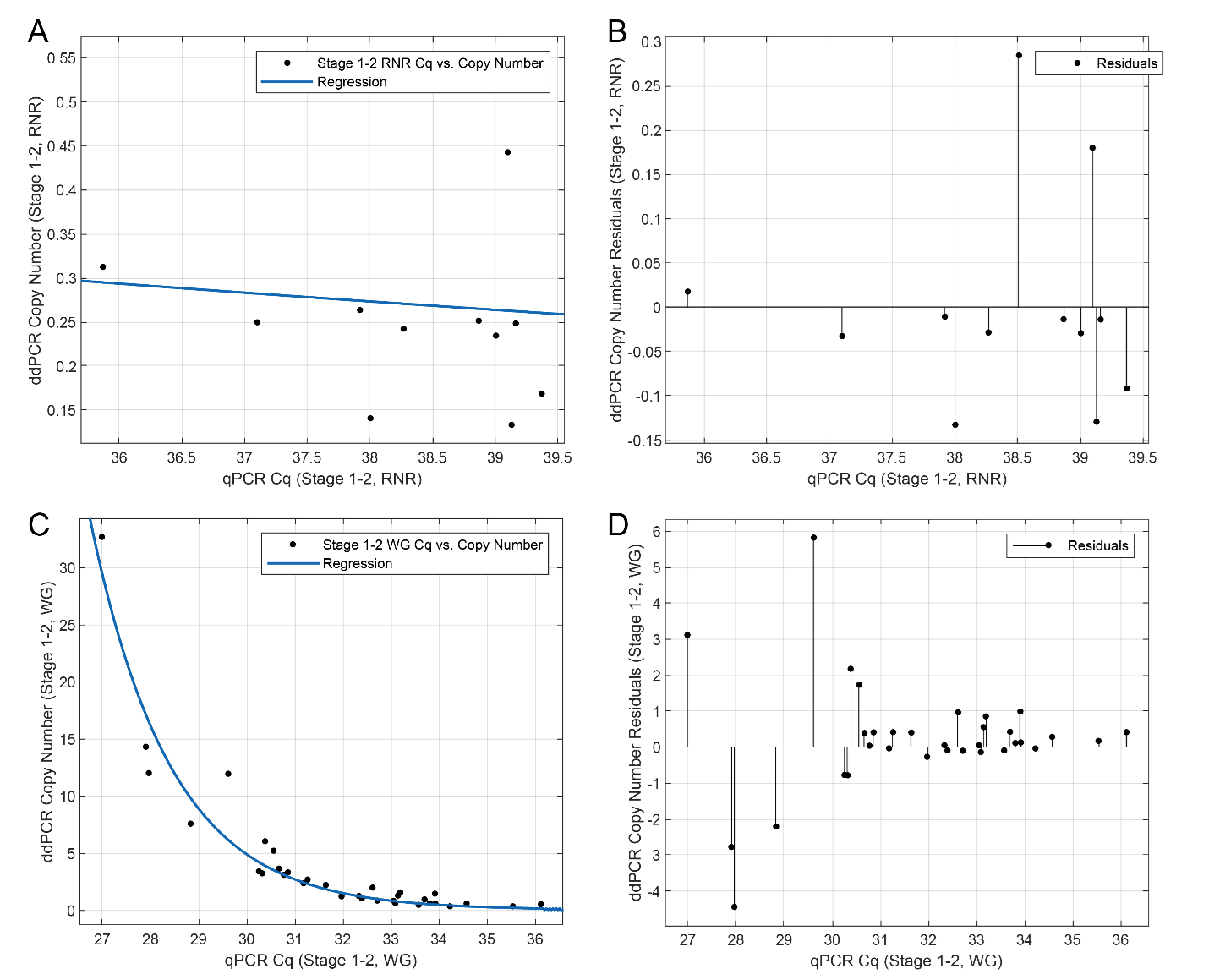

Supplement: S5 Fig — A. The Cq values and ddPCR copy number for 1st and 2nd nymphal stages based on RNR target amplification (black dots, n = 12). Regression curve fit to data, plotted as a solid blue line (R2 = 0.0071231). B. Residuals of regression in panel A with respect to Cq value. C. The qPCR Cq values and ddPCR copy numbers, respectively, for 1st and 2nd nymphal stages, based on WG target amplification (black dots, n = 33). The regression curve fit to data, plotted as a solid blue line (R2 = 0.92907). D. Residuals of regression in panel C with respect to Cq value. (TIF) [file pone.0323908.s005.tif]

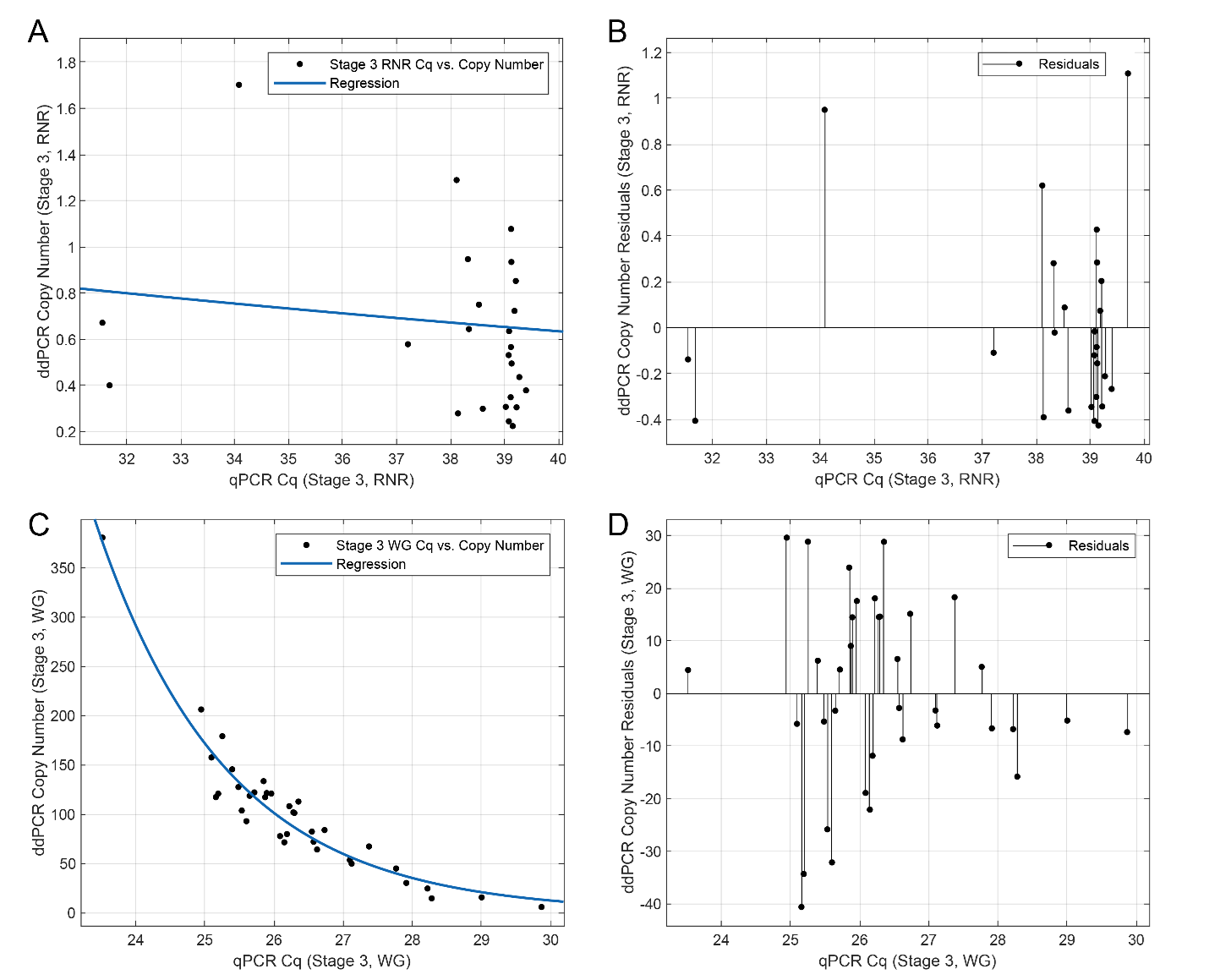

Supplement: S6 Fig — A. The qPCR Cq values and ddPCR copy numbers, respectively, for the 3rd nymphal stage based on RNR target amplification (black dots, n = 26). Regression curve fit to data, plotted as a solid blue line (R2 = 0.12791). B. Residuals of regression in panel A with respect to Cq value. C. The qPCR Cq values and ddPCR-copy numbers, respectively, for the 3rd nymphal stage, based on WG target amplification (black dots, n = 36). The regression curve fit to data, plotted as a solid blue line (R2 = 0.92635). D. Residuals of regression in panel C with respect to the Cq values. (TIF) [file pone.0323908.s006.tif]

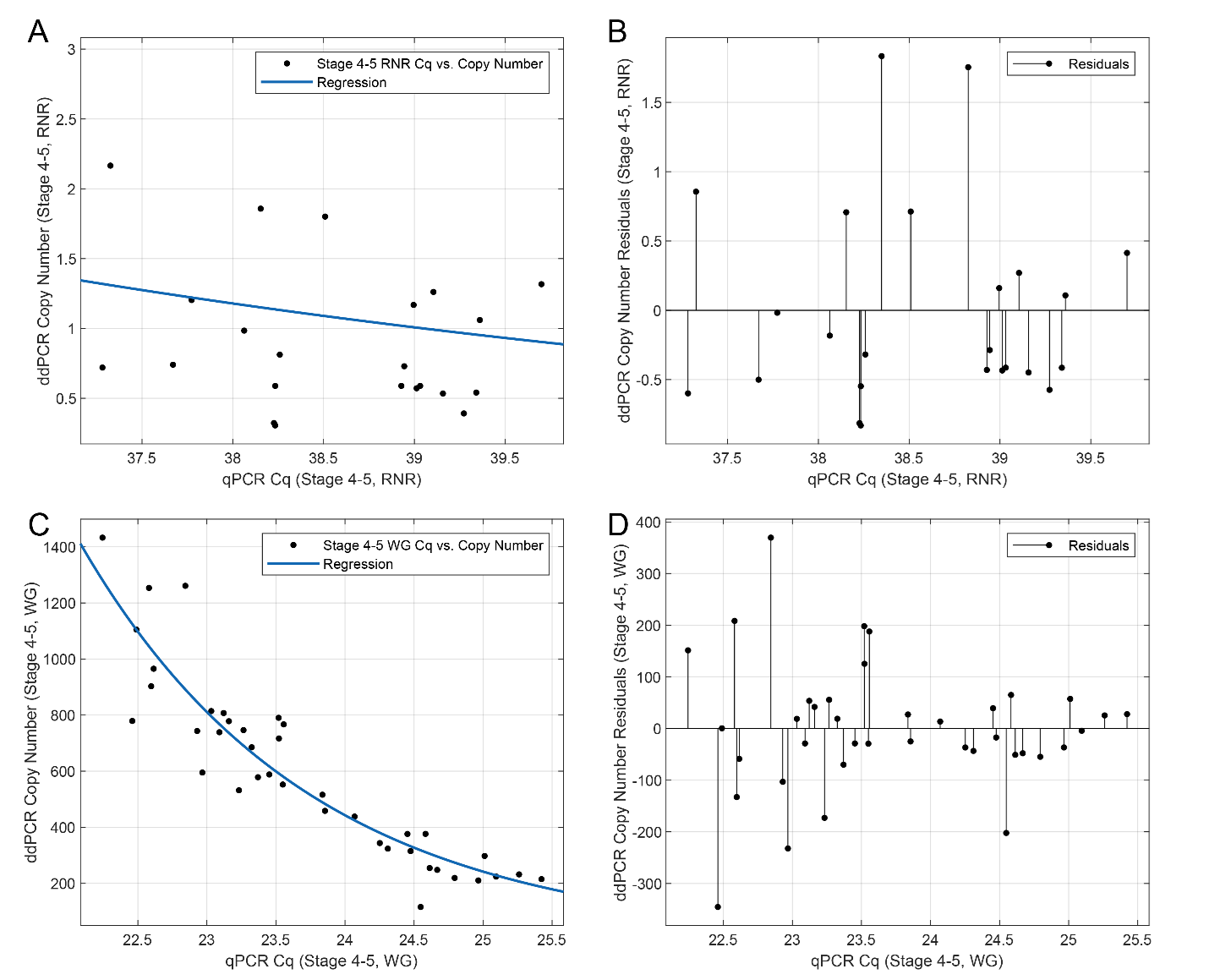

Supplement: S7 Fig — A. The Cq values and ddPCR copy numbers, respectively, for 4th and 5th nymphal stages based on RNR target amplification (black dots, n = 24). Regression curve fit to data is plotted as a solid blue line (R2 = 0.024877). B. Residuals of regression in panel A with respect to the Cq values. C. The qPCR Cq values and ddPCR-determined copy numbers, respectively, for the psyllid 4th and 5th nymphal stages, based on WG target amplification (black dots, n = 39). The regression curve fit to data is plotted as a solid blue line (R2 = 0.84816). D. Residuals of regression in panel C with respect to the Cq values. (TIF) [file pone.0323908.s007.tif]

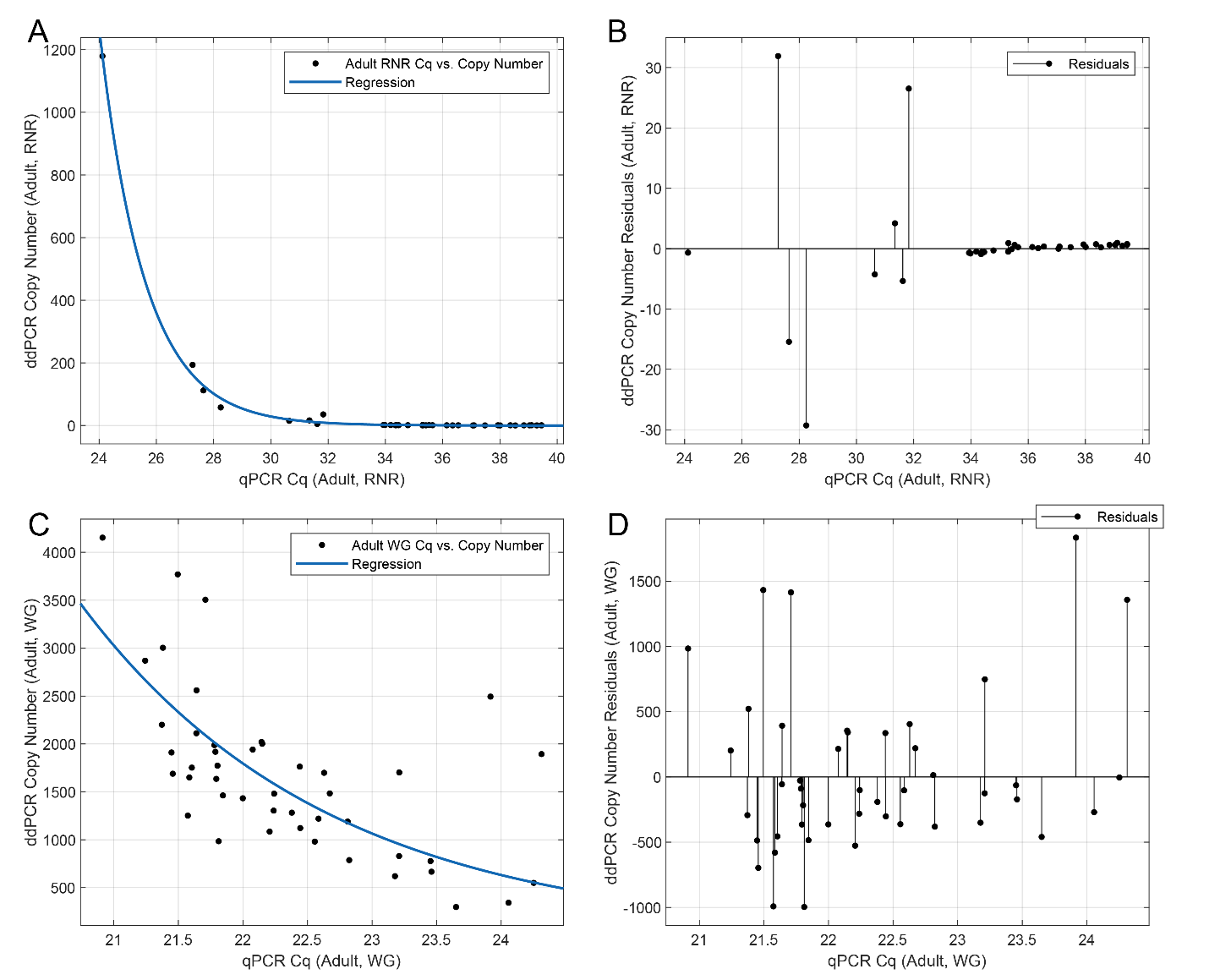

Supplement: S8 Fig — A. The Cq values and ddPCR copy numbers, respectively, for adult psyllids based on RNR target amplification (black dots, n = 36). Regression curve fit to data, plotted as a solid blue line (R2 = 0.99789). B. Residuals of regression in panel A with respect to the Cq values. C. The Cq values and ddPCR copy numbers, respectively, for adult psyllids based on WG target amplification (black dots, n = 45). Regression curve fit to data, plotted as a solid blue line (R2 = 0.46302). D. Residuals of regression in panel C with respect to the Cq values. (TIF) [file pone.0323908.s008.tif]
